# A 3D Vision-Based Localization Method for Automated Loading of Large-Sized Heat Exchangers

**DOI:** 10.3390/s26206616

**Published:** 2026-10-19

**Authors:** Zhenfeng Huang, Songxiao Cao, Sujuan Xiang, Dong Zhang, Tao Song, Zhipeng Xu, Qing Jiang

**Affiliations:** 1 College of Metrology, Measurement and Instrument, China Jiliang University, Hangzhou 310018, China; s24020804020@cjlu.edu.cn (Z.H.); songtao@cjlu.edu.cn (T.S.); xuzhipeng@cjlu.edu.cn (Z.X.); 06a0203051@cjlu.edu.cn (Q.J.); 2 Hangzhou Institute of Quality and Metrology, Hangzhou 310018, China; xiangsujuan@126.com (S.X.); zhangdong_1005@163.com (D.Z.)

**Keywords:** 3D vision-based localization, large-sized heat exchanger, incomplete point-cloud registration, automated loading

## Abstract

Manual loading of large-sized finned-tube heat exchangers has low productivity and imposes substantial physical demands on workers. In addition, industrial 3D vision systems often produce large regions of missing depth data when imaging highly reflective metallic surfaces, which complicates grasp-pose estimation for robot manipulators. Unlike randomly sparse measurements, these reflection-induced depth failures are spatially continuous and view-dependent: specular reflection may redirect the projected signal away from the receiver, producing invalid or erroneous depth values over substantial surface regions. Under such conditions, depth-completion methods may generate geometrically plausible but unverified estimates in unobserved regions, whereas correspondence-based registration becomes unreliable when valid overlap, stable local geometry, and distinctive features are simultaneously reduced. This paper develops a model-specific 3D vision-based localization method for automated loading of large-sized heat exchangers affected by structured reflection-induced point-cloud incompleteness. First, an industrial 3D camera synchronously acquires color and depth images. A hybrid interlayer-cardboard extraction strategy uses two-dimensional (2D) color-threshold morphological segmentation and three-dimensional (3D) back-projection as the primary branch, while activating a geometry-based point-cloud fallback when the color-based branch produces no valid candidate or returns a component count inconsistent with the production-task configuration. In 129 paired RGB and point-cloud samples, the color-based branch correctly processed 122 samples, and the fallback recovered the remaining seven cases under the evaluated production-line conditions. For product localization, the method integrates three complementary geometric operations: projected-area minimization for yaw estimation and lateral alignment, tail-feature constraints for head–tail determination and longitudinal anchoring, and inverse mapping of the complete CAD template to the observed product pose. This coordinated procedure exploits the remaining reliable global and regional geometry without reconstructing the missing surface, thereby reducing the pose-estimation bias caused by the shifted centroid of the incomplete point cloud. In the main registration experiment, the method was evaluated using 436 production-line samples with different stacking poses and levels of missing data, including severe cases with a missing rate greater than 30%. This 436-sample evaluation yielded an overall mean translation error of 5.21 mm, a nearest-neighbor RMSE of 1.98 mm, and an average registration-operator runtime of 531.03 ms per registration. By exploiting known product geometry and a reflection-resistant tail feature rather than relying on completed depth values, the proposed method provides deterministic localization for the evaluated heat-exchanger models and production-line conditions.

## 1. Introduction

With global climate change and economic development, air conditioners have become indispensable in modern buildings and daily life, driving sustained growth in the air-conditioning industry. In this context, the energy efficiency and performance of air-conditioning systems are of critical importance. As a key component of these systems, the heat exchanger directly affects cooling and heating performance. Among the numerous types of heat exchangers, the finned-tube heat exchanger (hereinafter referred to as the “heat exchanger”) is a highly efficient heat exchange device widely used in industrial and commercial fields. Owing to its high heat transfer efficiency, compact structure, and strong applicability, it is extensively used in heating, ventilation, and air-conditioning (HVAC) systems, industrial refrigeration, and transportation.

**Figure 1:**
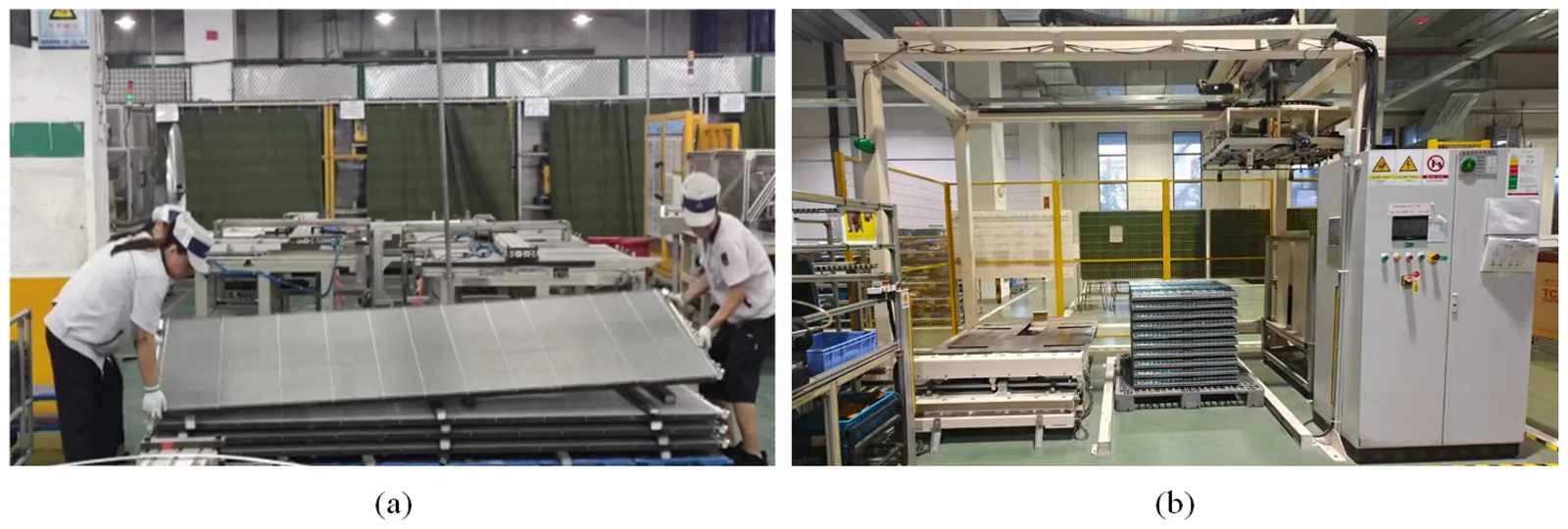
(**a**) manual loading; (**b**) automated loading.

To improve production efficiency, some HVAC manufacturers in China have adopted semi-automated approaches to complete the upstream welding and assembly processes of heat exchangers. However, the loading process, a critical step, still relies on manual labor, especially for large-sized heat exchangers with dimensions of 2300 mm × 1220 mm × 64.9 mm, 2300 mm × 1220 mm × 43.3 mm, or 2000 mm × 1220 mm × 43.3 mm. The largest models weigh more than 60 kg and typically require two to four workers. Such manual handling is not only labor-intensive and inefficient but also entails certain safety risks. Moreover, the fins and flat tubes of the heat exchangers are easily damaged during manual handling, which severely constrains the overall automation level and efficiency of the production line. Therefore, introducing an automated loading system to replace inefficient manual handling has become an urgent demand in the HVAC manufacturing field. Manual and automated loading are compared in Figure 1. However, during automation retrofitting, the highly reflective metallic surfaces of large-sized heat exchangers readily cause large regions of missing depth data in 3D vision systems, making pose estimation for the heat exchanger a technical bottleneck that restricts the deployment of the entire system. To this end, this paper proposes a 3D vision-based localization method for automated loading of large-sized heat exchangers, enabling reliable, fully automated grasping and loading of heat exchangers.

With the development of intelligent manufacturing, industrial robots have increasingly shifted from traditional 2D vision to 3D vision for unstructured grasping and automated loading. Limited by the lack of depth information, 2D vision struggles to handle workpiece stacking and spatial-pose variations, whereas 3D structured-light and time-of-flight (ToF) cameras provide the depth information required for multi-degree-of-freedom pose estimation in complex environments.

Numerous studies have proposed loading and unloading schemes combining machine vision with industrial robots. In the industrial field, Wang et al. [1] proposed a remote robotic automatic assembly system based on 3D visual guidance. Luo [2] adjusted the grasping positions for automated loading and unloading through visual recognition. Zheng et al. [3] applied robots to CNC machining, realizing automatic loading and unloading of workpieces. In recent years, these cutting-edge research achievements have been translated into practical applications by robot manufacturers. For example, through optimized design, the ABB IRB-1600 robot achieves a cycle time reduction of approximately 50% compared with similar robots in material handling and loading/unloading operations. FANUC has also launched automated machine-tool loading/unloading solutions integrated with robot vision systems. By precisely identifying and localizing workpieces, these solutions optimize the entire machining process and further improve the efficiency and reliability of automated production.

However, industrial 3D vision systems remain challenged by large-sized heat exchangers with metal fins, highly reflective aluminum foil, and multilayer stacked structures. For active RGB-D and structured-light cameras, reflected illumination from a specular surface may be redirected away from the receiver rather than returning along a measurable path. Consequently, valid depth can be obtained only from limited surface regions with favorable viewing geometry, while other regions may exhibit invalid measurements, depth holes, or erroneous depth values [4,5]. In structured-light measurement, strong reflection can also saturate the imaging sensor and corrupt phase recovery, resulting in the loss of critical surface information [6,7,8]. Therefore, the missing data on reflective heat exchangers are not random sparse samples; they are material-, illumination-, and viewpoint-dependent continuous regions that can substantially disrupt the geometric integrity of the acquired point cloud. Specular surfaces may also produce spatially structured false returns rather than only missing measurements. Zhu et al. used laser-return intensity and mirror symmetry to identify mirror-induced measurements and correct the corresponding point-cloud and occupancy-grid representations [9]. Their results further demonstrate that reflection-related point-cloud degradation should be distinguished from ordinary independent measurement noise.

To address large missing regions, existing research has focused on depth-completion techniques at the data level. Traditional optimization methods have demonstrated their ability to restore missing depth values for complex geometries; for example, Satapathy et al. [10] proposed a robust algorithm based on superpixels and a non-local Gauss–Markov random field (GMRF) prior, which effectively fills large regions of missing depth data, while Ku et al. [11] proposed a lightweight and fast completion method deployable on the CPU, achieving real-time restoration of sparse LiDAR data.

In recent years, deep learning methods, owing to their powerful feature fitting capabilities, have become dominant in the field of depth completion. Wan et al. [12] proposed a network based on hierarchical feature reconstruction and dynamic weight sampling that generates high-density depth maps under the guidance of color images. Sun et al. [13] introduced a multi-scale spatial propagation module and tensor decomposition, expanding the propagation range of depth information while reducing feature redundancy. Zhang et al. [14] constructed a self-ensembling framework based on density-aware consistency, significantly improving the generalization ability by leveraging teacher–student models and unlabeled data. Furthermore, for cross-scenario applications, Xiao et al. [15] constructed a domain-shared confidence domain, achieving sensor data fusion under unsupervised conditions.

Although these completion methods have demonstrated effectiveness for sparse LiDAR measurements, random holes, occlusion boundaries, and standard RGB-D scenes, their assumptions do not fully match the present industrial setting. In the heat-exchanger scenario, missing observations are concentrated on highly reflective fin regions and are coupled with surface orientation, exposure, and viewing direction. Such large, spatially continuous invalid regions may cross repeated fins, stepped edges, and geometric boundaries, for which local smoothness, color guidance, or spatial propagation cannot guarantee preservation of true surface discontinuities. In addition, specular highlights may vary with illumination and viewpoint, such that RGB appearance does not always provide a stable geometric cue for depth restoration [5,16].

Learning-based completion methods further depend on the availability of training data that represent the sensor configuration, material properties, object geometry, and missing-data distribution encountered during deployment. Models trained predominantly on road scenes, indoor environments, or commonly observed objects may not adequately represent the reflection pattern of large aluminum-foil heat exchangers with dense repetitive fins. When large regions are unobserved, a network may generate geometrically plausible but unverified depth estimates; such estimates cannot be directly treated as reliable physical constraints for collision-sensitive robot grasping and path planning. Moreover, many dedicated solutions for reflective surfaces rely on additional sensing configurations, such as multi-exposure acquisition, multi-view imaging, or polarization hardware, which increase calibration and deployment complexity in an existing production line [8,16].

Recent RGB-D perception studies have explored how depth-derived geometry can complement appearance information in semantic understanding. RCNet employs two specialized networks with shared RGB-D inputs: one extracts orientation-sensitive features in the spatial domain, while the other aggregates macrostructural features in the frequency domain; mutual learning coordinates their complementary representations [17]. Priors Meet Hindsight uses an RGB-D teacher network to inject depth-derived geometric priors into a lightweight RGB student network during training, enabling RGB-only inference [18]. For indoor semantic parsing, Unify then Align combines semantic and geometric feature streams and uses adaptive deformable fusion to reduce their pixel-level spatial misalignment [19]. These studies show that geometric cues can improve semantic perception when appearance information alone is ambiguous. However, they address defect detection or semantic parsing in image and feature spaces rather than estimating the rigid transformation between incomplete three-dimensional point sets.

Local iterative registration methods estimate the rigid transformation from geometric proximity. ICP is sensitive to initialization, incorrect nearest-neighbor correspondences, and insufficient overlap, whereas GICP additionally depends on stable local covariance and surface-geometry estimates [20,21]. Reflection-induced missing regions can therefore degrade both methods by leaving sparse or truncated neighborhoods and by shifting the observed geometric center away from that of the complete template.

Global correspondence-based methods reduce dependence on initialization by matching local features or geometric configurations. FPFH–RANSAC uses local descriptors and consensus sampling, Fast Global Registration optimizes a robust correspondence objective, Super4PCS searches congruent point sets, and TEASER++ estimates a certifiable transformation under extreme correspondence outliers [22,23,24,25]. These approaches are effective when sufficiently distinctive and spatially distributed correspondences remain. For the products considered here, however, repeated fins generate locally similar structures, while continuous reflection-induced missingness reduces valid overlap and removes entire groups of potentially discriminative features. The resulting ambiguous or poorly distributed correspondences can destabilize both global hypothesis generation and subsequent local refinement.

For rigid registration under incomplete or low-overlap observations, recent learning-based methods have improved correspondence extraction and consistency verification. Deep Global Registration and PointDSC suppress unreliable matches through learned inlier estimation and spatial-consistency reasoning, while PREDATOR, CoFiNet, and GeoTransformer explicitly improve correspondence extraction in overlapping regions [26,27,28,29,30]. Nevertheless, their performance still depends on the presence of informative overlap and on training data that represent the deployment geometry, sensing noise, and missing-data pattern. Large structured gaps and repetitive metallic fins can simultaneously reduce the observable overlap and weaken feature distinctiveness, creating a domain condition that is not well represented by common indoor or object registration benchmarks.

Industrial registration studies have introduced robust estimation and geometric constraints for specific operating conditions. Han et al. combined MSAC with GICP for tee-pipe registration [31]; Xu et al. proposed an ISS–FPFH–RANSAC initialization followed by robust ICP for industrial components [32]; Su et al. used a locally consistent Gaussian mixture model for robotic inspection [33]; and Agarwal et al. incorporated local shape constraints to improve registration with missing regions and structural outliers [34]. These studies show that industrial perception and registration can benefit from robust estimation and task-specific geometric constraints. However, rigid registration of known industrial components remains challenging when continuous reflection-induced missingness coincides with repetitive local geometry and limited distinctive correspondences.

Accordingly, the main challenge in this application is to estimate the pose of a known industrial component when the missing measurements are extensive, structured, and concentrated on repetitive reflective surfaces. The proposed method is a model-specific point-cloud registration strategy for incomplete observations in a fixed industrial production environment. Within this scope, the methodological contribution is the coordinated use of projected-area alignment, tail-constrained orientation and longitudinal anchoring, and inverse CAD-template mapping to exploit the remaining reliable geometry of the incomplete observation. The main contributions of this study are summarized as follows.

1. A model-specific geometric registration strategy is developed for structured incomplete point clouds of known heat-exchanger models. It integrates projected-area minimization for yaw estimation and lateral alignment with tail-region depth constraints for head–tail determination and longitudinal anchoring, reducing dependence on distinctive local descriptors and densely distributed correspondences.
2. The accumulated rigid transformation is inverted and applied to the complete CAD-template point cloud, mapping the template directly to the observed product pose. This operation provides geometrically complete data for grasp-pose calculation while avoiding depth completion and reducing the localization bias caused by the shifted centroid of the incomplete scan.
3. The integrated strategy is evaluated using 436 production-line samples covering multiple heat-exchanger models, stacking configurations, and missing-data levels. Its performance within the investigated operating conditions is further examined through component-level ablation, controlled tail-region degradation, parameter-sensitivity analysis, comparisons with representative registration methods, evaluation on an additional production-line product variant, and end-to-end robotic loading experiments.

## 2. Vision System Design and Methodology

### 2.1. Vision System Design

**Figure 2:**
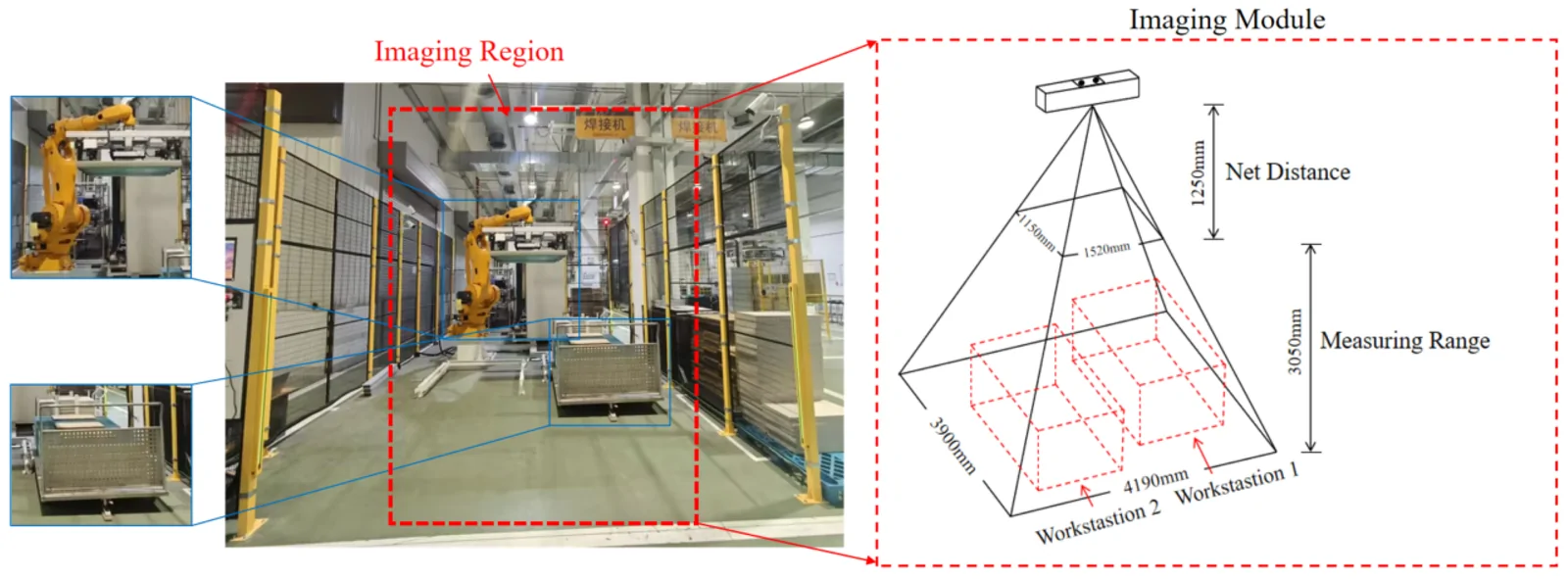
3D camera imaging system.

Figure 2 illustrates the 3D camera imaging system used in the proposed method. In the production scenario, the maximum stacking height of the products is approximately 1.3 m. To cover the working area, the RGB-D camera is mounted at a height of 4.4 m above the ground, acquiring 3D spatial information by capturing the top of the stack in real time. The system employs the PM806-E1 camera produced by Tuyang Technology, which integrates an infrared laser projector, an infrared camera, and an RGB camera. Its imaging principle is to project an infrared laser beam and capture the reflected light with the infrared camera, from which the depth data are computed based on phase information. The camera achieves a measurement accuracy of 3.4 mm at a distance of 4300 mm, a depth image resolution of 1280 × 960 pixels, and a field of view of 4190 mm × 3900 mm at the working distance. The camera SDK converts the acquired raw depth data into 3D point clouds for subsequent processing.

**Figure 3:**
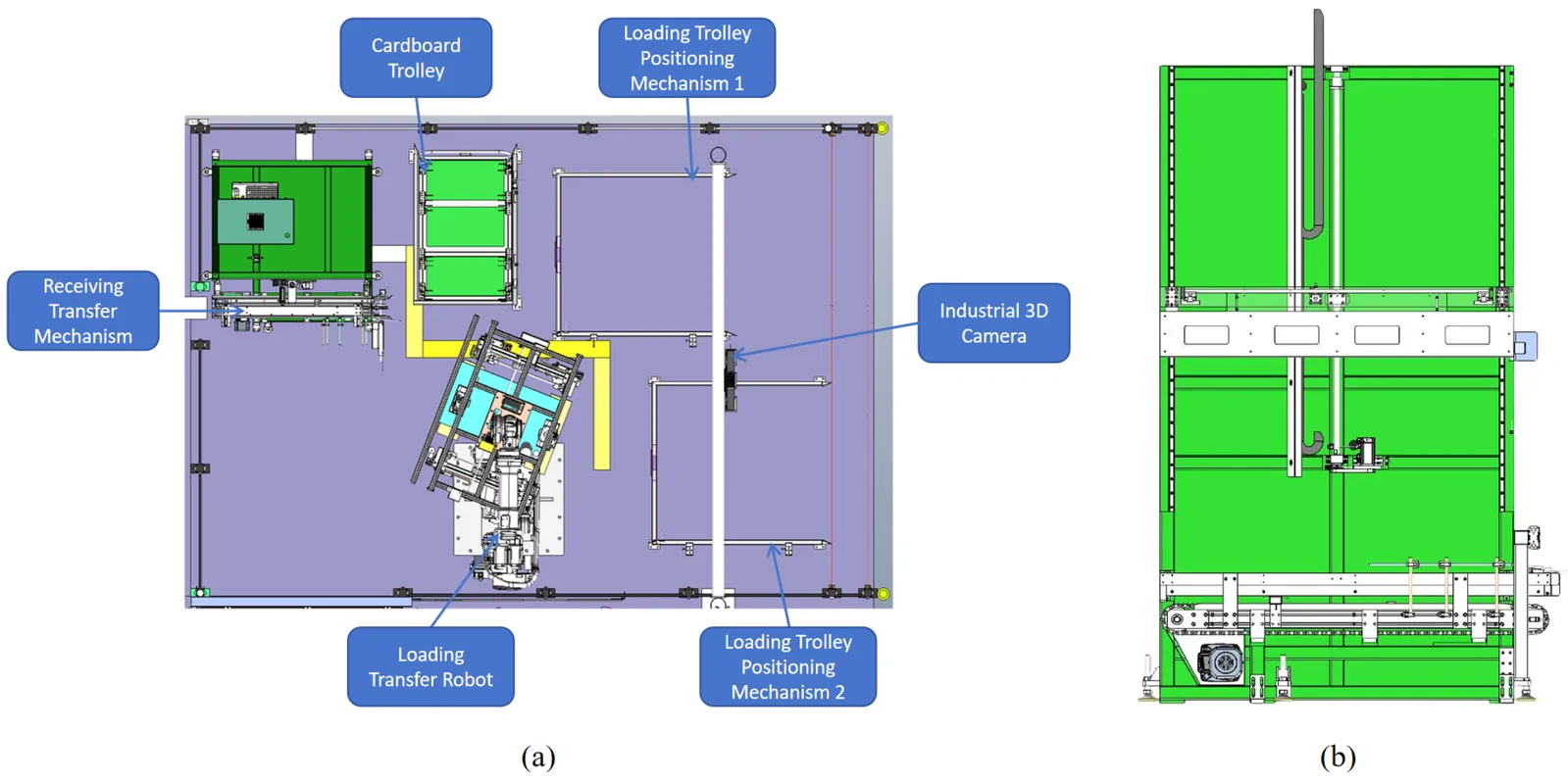
Layout and mechanical configuration of the loading system: (**a**) layout of the loading area; (**b**) enlarged view of the receiving transfer mechanism.

**Figure 4:**
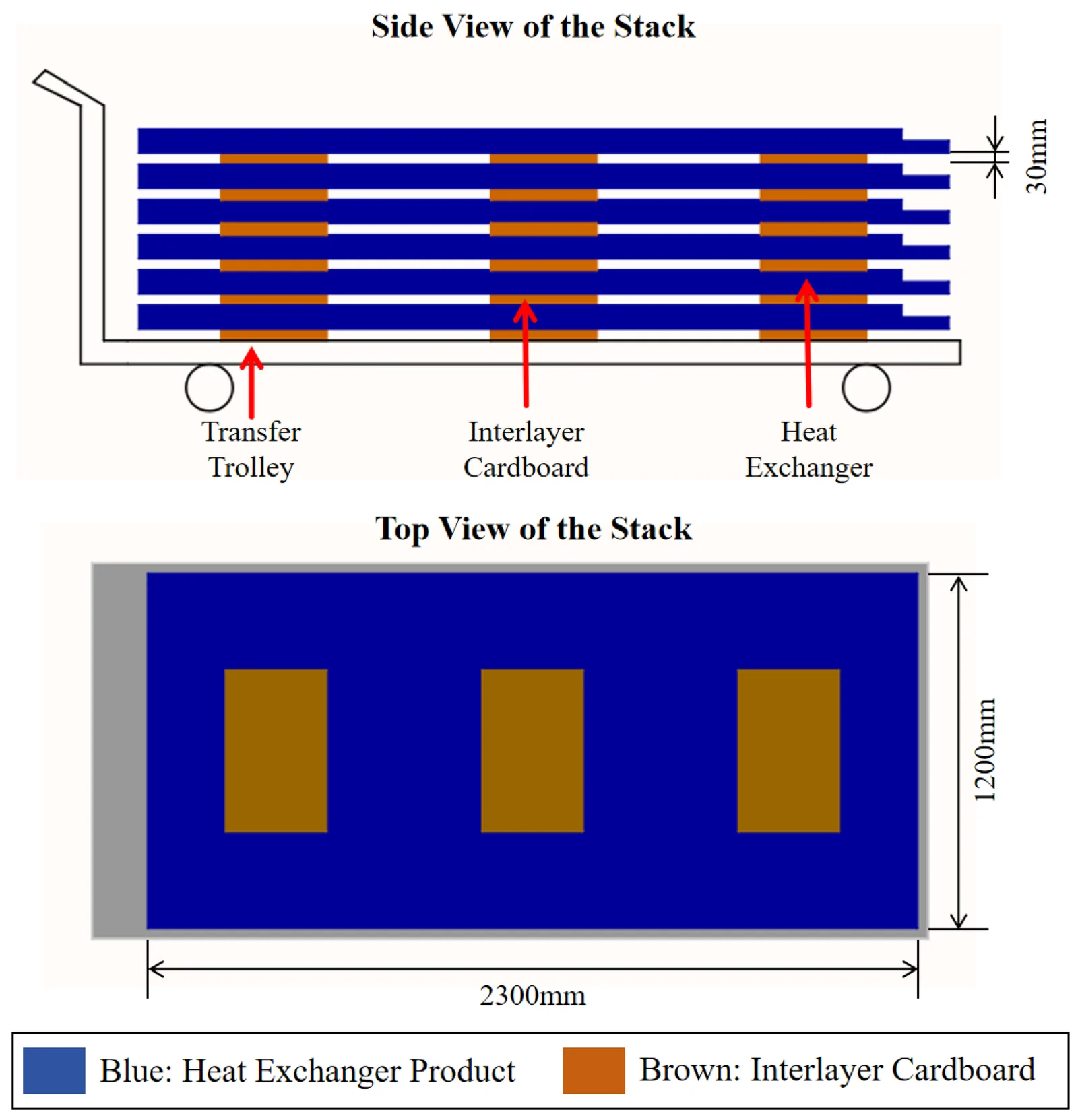
Schematic diagram of heat-exchanger stacking and nominal separator clearance.

**Figure 5:**
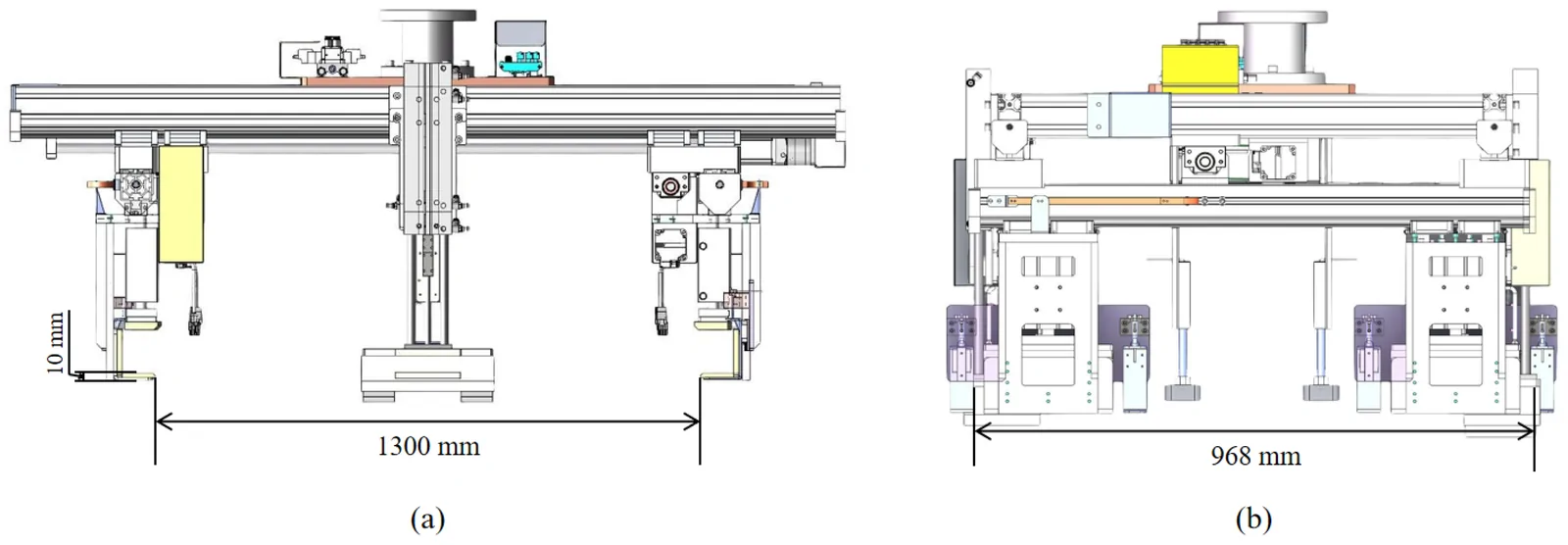
Mechanical configuration and dimensions of the gripper assembly: (**a**) front view; (**b**) side view.

Figure 3 shows the layout of the loading area and the receiving transfer mechanism. Figure 4 shows the typical stacking pattern of large-sized heat exchangers. The stack comprises multiple layers supported by a transfer trolley. To prevent collision and wear between metal components, two or three standardized compliant separators (cardboard or foam board) are placed between adjacent heat exchangers. Figure 5 presents front and side views of the gripper assembly. The 1300 mm dimension in Figure 5a denotes the maximum effective opening of the fully opened gripper, and the 10 mm dimension denotes the gripper-finger thickness. The 968 mm dimension in Figure 5b denotes the overall width of the gripper. The permissible localization errors were determined from the minimum nominal clearances between the product, the receiving transfer mechanism, and the end-effector gripper. The maximum nominal product width is 1220 mm, giving a lateral clearance of 40 mm on each side under centered grasping. The receiving transfer mechanism shown in Figure 3b provides a maximum effective longitudinal accommodation length of 2400 mm. With a maximum product length of 2300 mm, the nominal clearance is 50 mm at each longitudinal end. Finally, the nominal interlayer cardboard thickness is 30 mm. Centered insertion of the 10 mm gripper finger therefore provides a theoretical vertical half-clearance of 10 mm. These nominal clearances can be summarized as

$$C_{x}=\frac{1300-1220}{2}=40\;\mathrm{mm},\;C_{y}=\frac{2400-2300}{2}=50\;\mathrm{mm},\;C_{z}=\frac{30-10}{2}=10\;\mathrm{mm}.$$

The nominal clearances above are geometric upper bounds rather than permissible vision errors. To retain margins for robot positioning uncertainty, gripper assembly error, structural compliance, stacking variation, and yaw-induced end displacement, the localization requirements were conservatively set to $|e_{x}|\leq 10\;\mathrm{mm}$, $|e_{y}|\leq 15\;\mathrm{mm}$, and $|e_{z}|<10\;\mathrm{mm}$. The X- and Y-direction limits retain nominal residual margins of 30 mm and 35 mm, respectively. For a product of length $L_{p}$, a yaw error Δψ additionally produces an approximate lateral end displacement $d_{\psi }=\left(L_{p}/2\right)\sin\left|\Delta \psi \right|$; this displacement is included in the reserved X-direction clearance. Because the Z-direction value is the theoretical centered-insertion limit, the actual vertical error must remain below 10 mm rather than consume the full clearance. During automated handling, the robot first removes the interlayer cardboard using a vacuum-cup end effector and then grasps the heat exchanger with the gripper to avoid surface damage caused by cardboard slippage.

**Figure 6:**
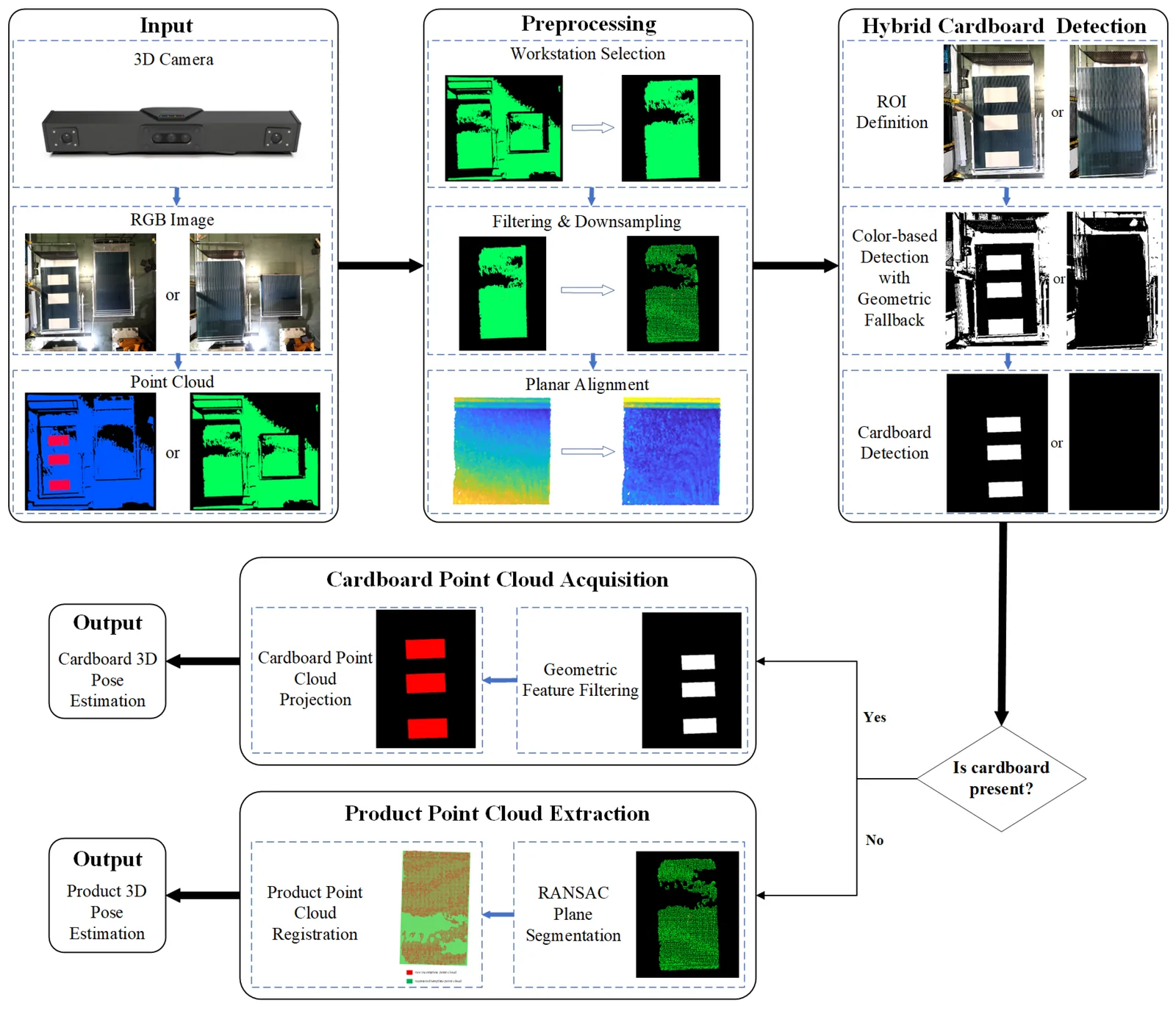
Overall flowchart of the proposed vision algorithm.

The overall workflow is shown in Figure 6. After the camera synchronized acquisition of the RGB image, depth image, and raw point cloud, the station ROI is preprocessed and corrected. The system then determines whether the uppermost target is interlayer cardboard or a heat exchanger and invokes the corresponding extraction and pose-estimation branch. The estimated pose is transmitted to the robot controller only after it passes the station-specific safety-validity check; otherwise, robot motion is inhibited and scene reacquisition is triggered.

### 2.2. Object Detection and Localization Based on 3D Vision

#### 2.2.1. Point Cloud Preprocessing and Correction

To support the simultaneous operation of multiple industrial stations, the system uses a large-field-of-view camera to cover two work areas. However, wide-area imaging inevitably captures substantial environmental background noise together with information from the target station. The method first defines a workstation-specific Region of Interest (ROI) using directional filtering to spatially constrain the raw point cloud and remove invalid outliers outside the workspace. This step suppresses environmental interference while reducing the amount of data to be processed. The point cloud within the region of interest (ROI) is then downsampled and filtered with a grid size of 8 mm to remove outliers and reduce data volume, thereby improving the speed of subsequent processing. Finally, the point cloud within the ROI is corrected to establish a consistent reference plane.

**Figure 7:**
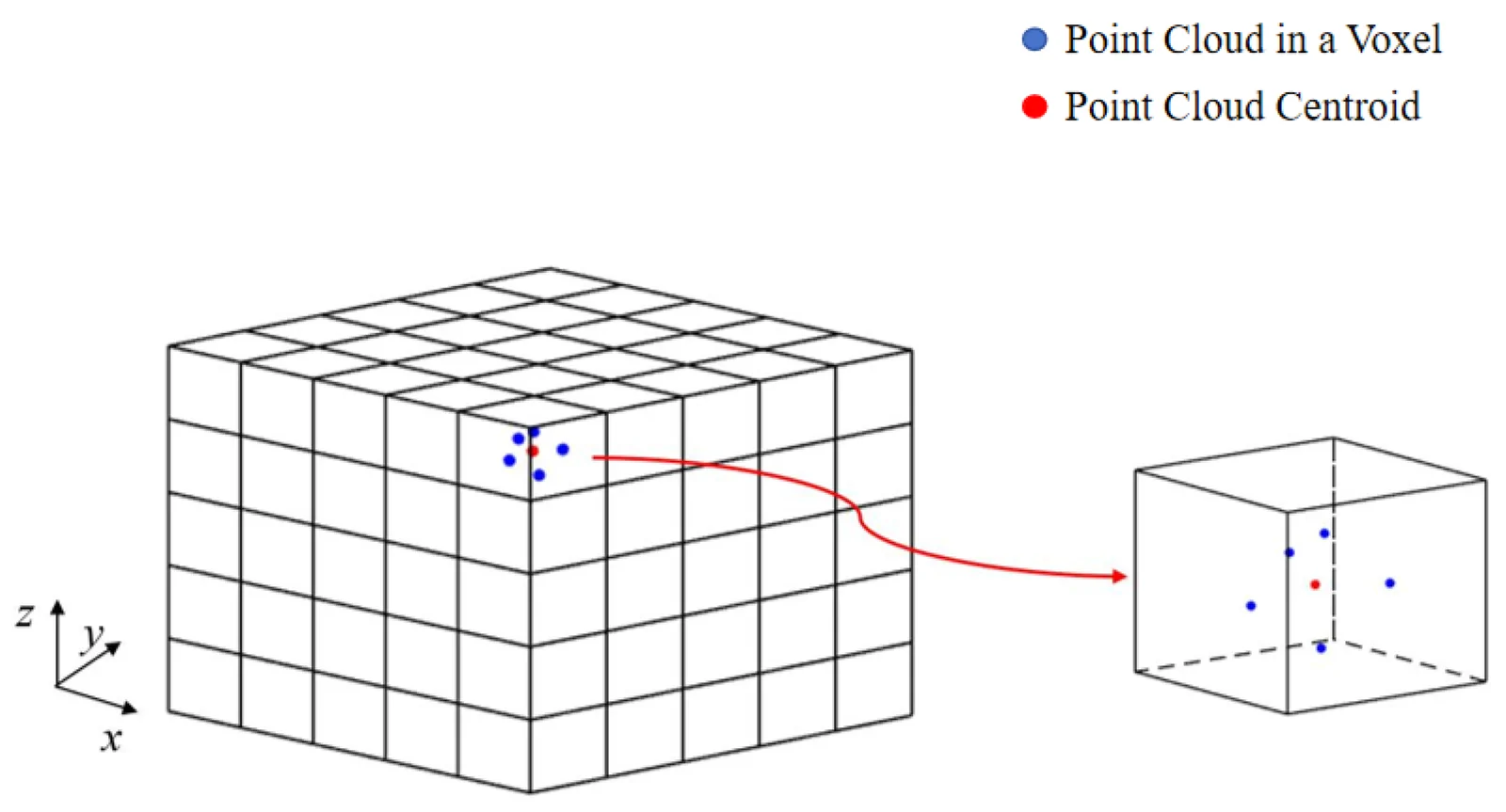
Illustration of voxel-grid downsampling.

Voxel-grid downsampling was selected to reduce the data volume while preserving a spatially uniform representation. As illustrated in Figure 7, the three-dimensional space is divided into cubic voxels, and the centroid of each occupied voxel is retained. For a voxel containing *n* raw points, where the *i*-th point is $P_{i}={\left[x_{i},y_{i},z_{i}\right]}^{T}$, the representative centroid $P_{c}$ is

(1) $$P_{c}={\left[\frac{1}{n}\sum_{i=1}^{n}x_{i},\frac{1}{n}\sum_{i=1}^{n}y_{i},\frac{1}{n}\sum_{i=1}^{n}z_{i}\right]}^{T}$$

**Figure 8:**
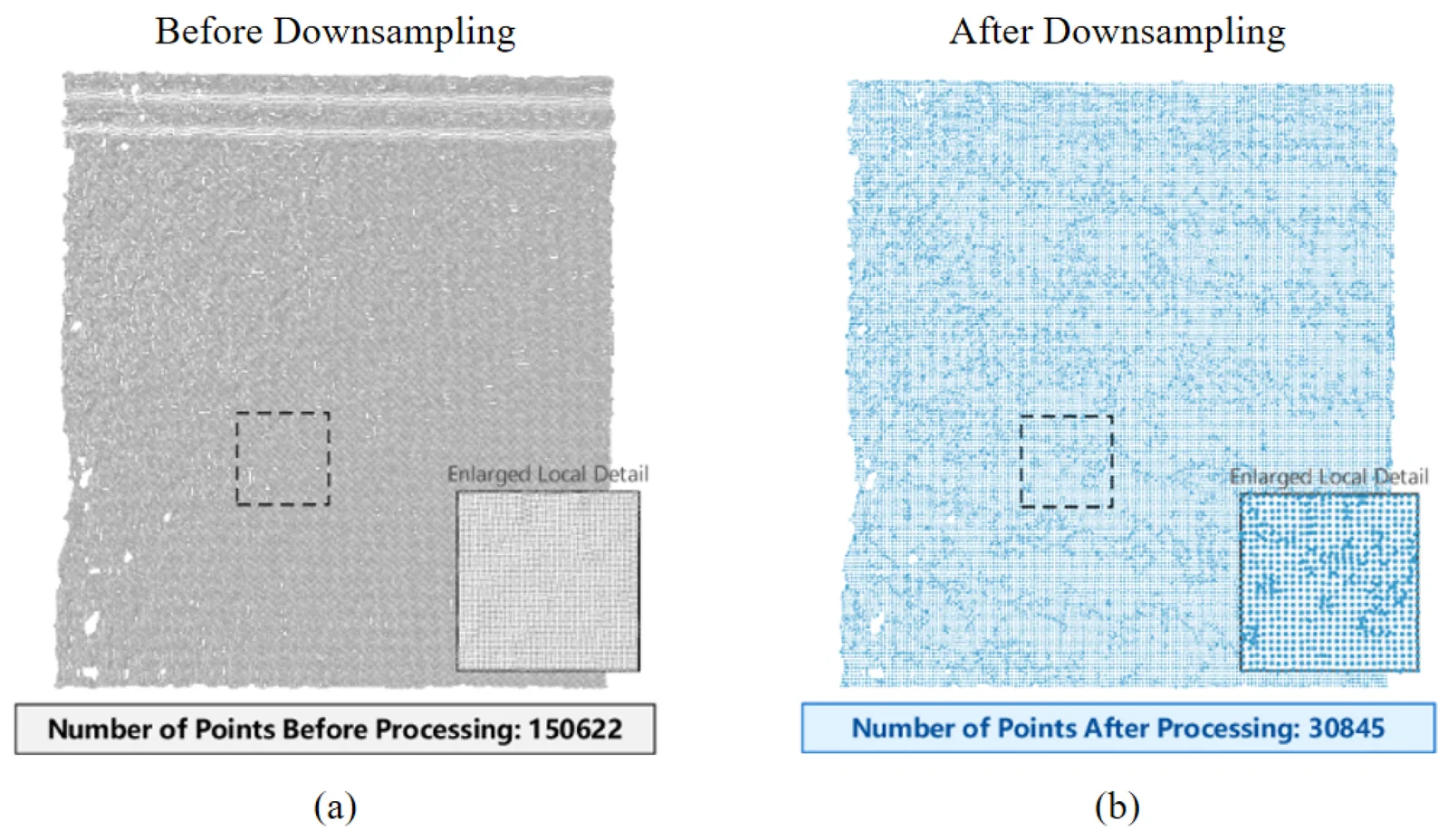
Point clouds before and after downsampling: (**a**) raw product point cloud; (**b**) point cloud after voxel-grid downsampling.

The reduction in point count while retaining the principal product geometry is illustrated in Figure 8.

**Figure 9:**
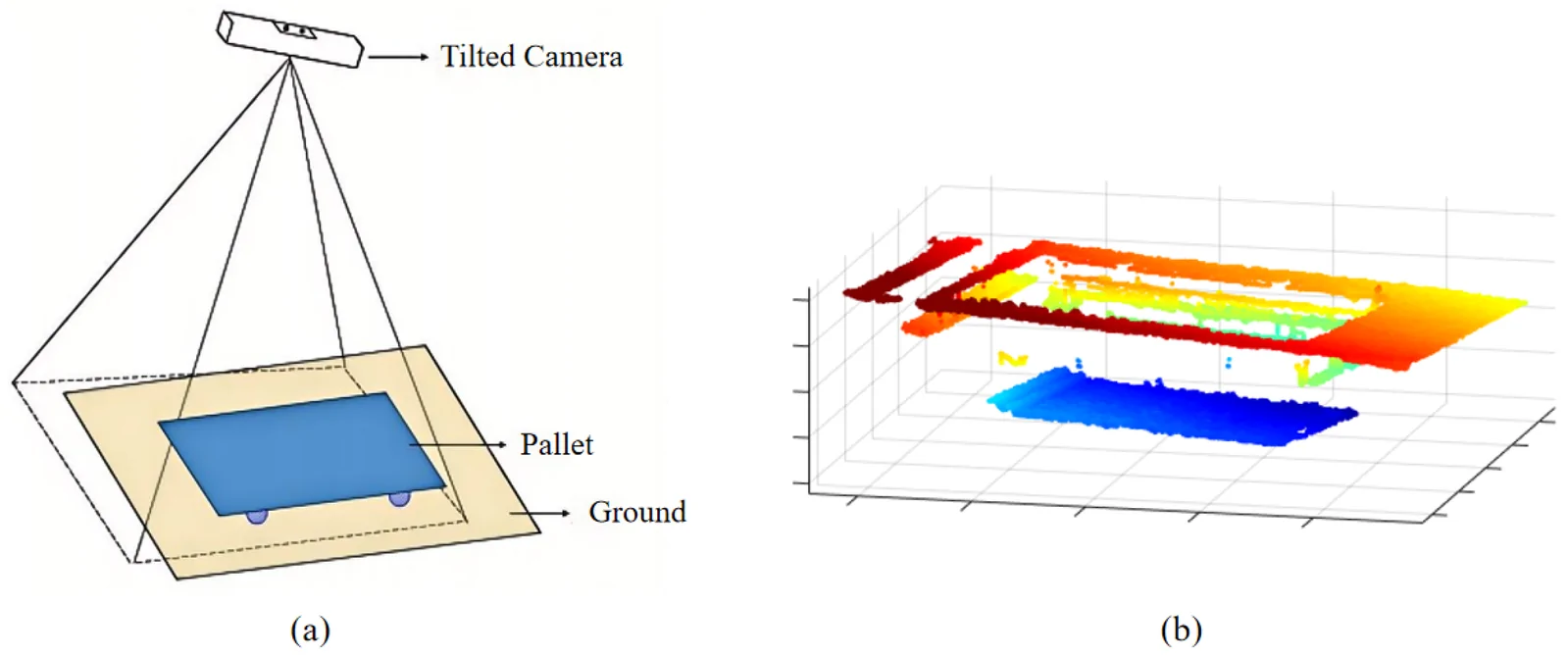
Camera installation and the raw tilted point cloud: (**a**) camera installation; (**b**) raw tilted point cloud in the camera coordinate system before correction.

Residual roll and pitch deviations in the installed camera cause the stack surface to appear tilted in the camera coordinate system, as shown in Figure 9. A one-time reference-plane correction is therefore performed during system commissioning, and the resulting rotation matrix is kept fixed throughout the subsequent production experiments.

The pallet surface is used as the physical reference plane and is acquired once during system commissioning. Because the ROI may contain non-plane points and noise, Random Sample Consensus (RANSAC) is used to obtain an outlier-resistant plane estimate. The procedure is as follows.

First, three non-collinear points, $S_{1}\left(x_{1},y_{1},z_{1}\right)$, $S_{2}\left(x_{2},y_{2},z_{2}\right)$, $S_{3}\left(x_{3},y_{3},z_{3}\right)$, are randomly selected from the initial point-cloud set *S* and substituted into the plane equation in Equation (2) to obtain the plane-model parameters for the current iteration.

(2) Ax+By+Cz+D=0,C≠0

After the plane-model parameters have been calculated, the remaining points in *S* are examined, and the spatial distance $d_{i}$ from each point to the current plane is computed. If $d_{i}$ satisfies the inequality in Equation (3), the point is identified as an inlier of the current plane.

(3) $$\frac{\left|Ax_{i}+By_{i}+Cz_{i}+D\right|}{\sqrt{A^{2}+B^{2}+C^{2}}}<t$$

The random sampling and verification process is then repeated. If the number of inliers in the current iteration exceeds that of all previous iterations, the corresponding plane-fitting parameters are retained. After a preset number of iterations, or when the inlier ratio stabilizes, the resulting plane is selected as the best-fitting plane. In the implementation, the RANSAC point-to-plane distance threshold was set to 10 mm. After the plane inliers had been identified by RANSAC, all inlier points were refitted by total least squares to obtain a refined plane normal.

The resulting refined unit normal vector of the pallet surface is denoted by $n_{ground}={\left(A,B,C\right)}^{T}$, which represents the spatial orientation of the pallet in the current camera coordinate system.

Let $v_{z}={\left(0,0,1\right)}^{T}$ denote the camera Z-axis. The correction angle θ between $n_{ground}$ and $v_{z}$ is calculated using Equation (4), and the corresponding rotation matrix *R* aligns the reference-plane normal with the Z-axis.

(4) $$\theta =\arccos\left(\frac{n_{ground}\cdot v_{z}}{∥n_{ground}∥∥v_{z}∥}\right)$$

**Figure 10:**
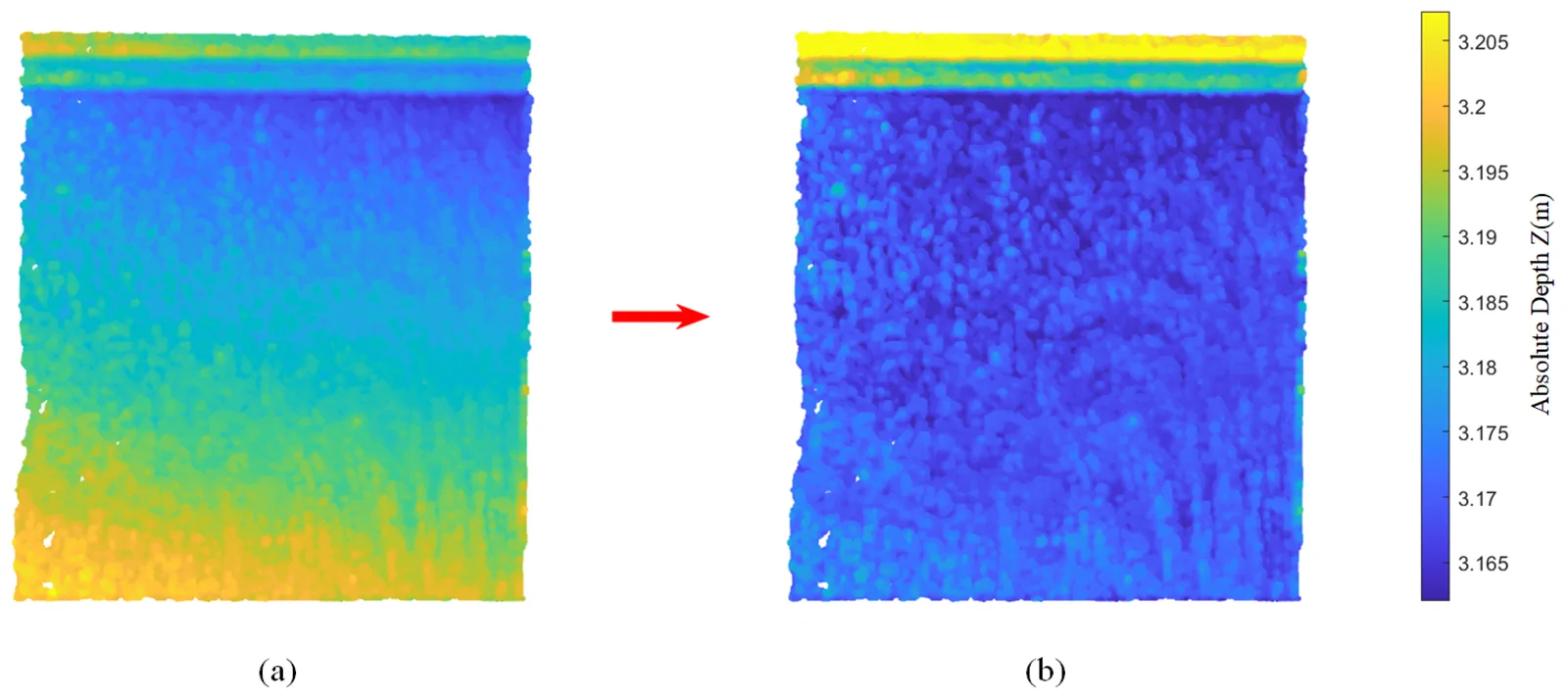
Depth distribution of the product surface before and after correction: (**a**) before correction; (**b**) after correction. the arrow indicates the transition from before to after correction.

After applying *R*, the product surface becomes approximately parallel to the XOY plane, as shown in Figure 10, providing a common geometric reference for subsequent segmentation and pose estimation.

#### 2.2.2. Interlayer Cardboard Recognition and Point Cloud Extraction

**Figure 11:**
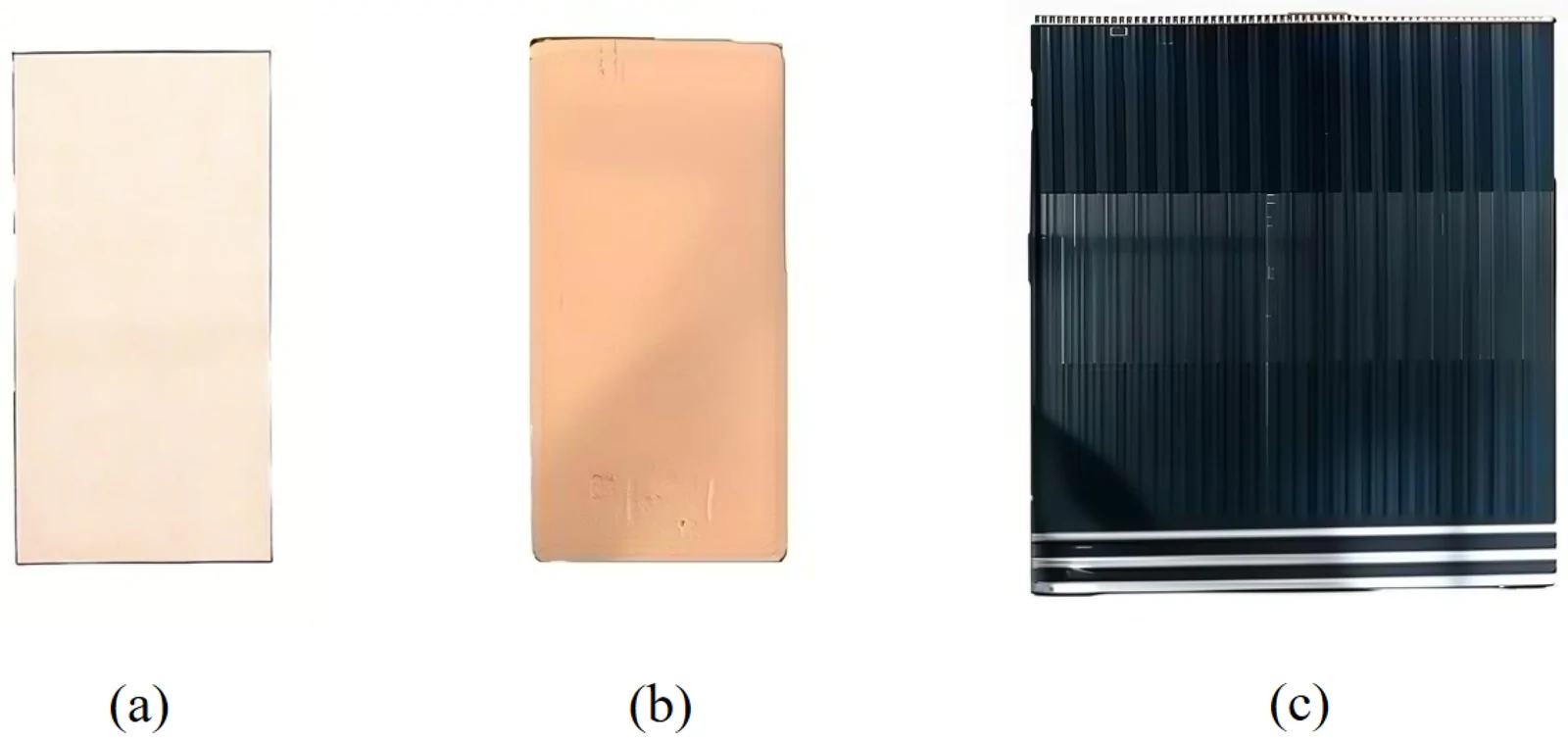
Interlayer cardboard and the product: (**a**) white cardboard; (**b**) brown cardboard; (**c**) dark product.

Given the distribution of points on the stack surface within the camera’s field of view, extracting the interlayer cardboard presents the following technical challenges. First, the cardboard contains far fewer points than the heat exchanger or transfer trolley, resulting in low target-to-background contrast. Second, the nominal cardboard thickness is 30 mm, whereas its measured thickness under production conditions is typically 25–28 mm. Given the camera mounting height and single-point measurement variation along the Z-axis, geometric segmentation methods alone, such as bounding-box segmentation or RANSAC plane fitting, can result in under-segmentation or detection failure. Industrial 3D cameras can simultaneously acquire high-resolution RGB images and depth data, and their intrinsic and extrinsic parameters are available from factory calibration. As shown in Figure 11, the interlayer cardboard differs substantially in color from the heat exchanger. The proposed method therefore adopts a hybrid recognition strategy. Its primary branch combines color-space threshold segmentation with 3D back-projection to efficiently extract the cardboard point cloud under the calibrated station conditions. When the color-processing branch does not produce a valid candidate, an independent geometry-based fallback operates directly on the corrected point cloud.

**Figure 12:**
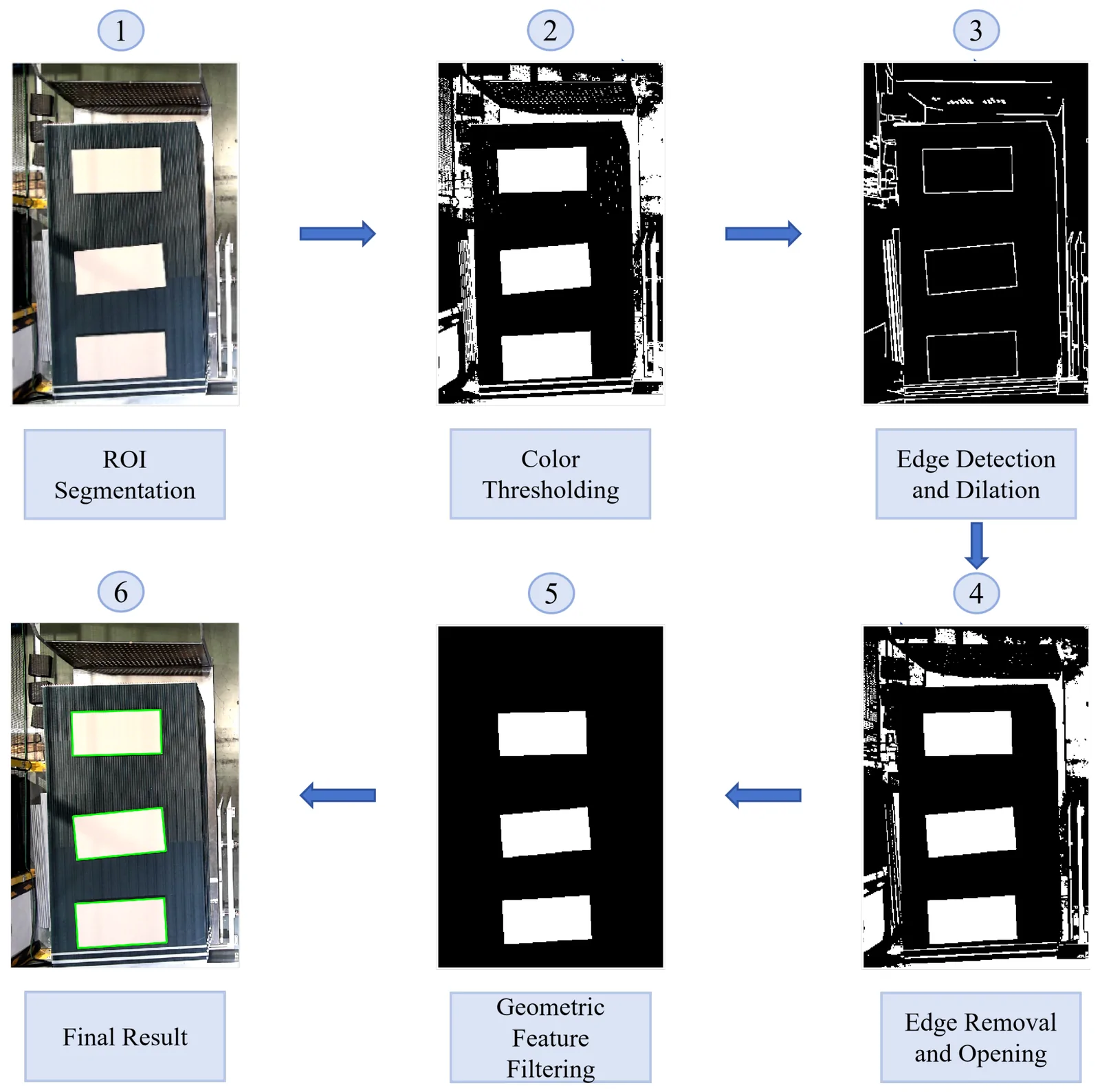
Color-based primary branch for interlayer-cardboard recognition: the arrows and numbers indicate the sequence of the algorithm steps.

Figure 12 shows the 2D cardboard-mask generation process of the color-based primary branch. First, the RGB image acquired by the industrial 3D camera is segmented into a Region of Interest (ROI) for the target station. This reduces the image-processing area and computational cost, while suppressing interference from same-colored features in non-target areas within the field of view.

Within the ROI, image pixels are filtered according to preset color thresholds, and candidate pixels for the target cardboard are extracted to generate the initial color-constrained mask, as shown in Step 2 of Figure 12. To prevent undesired connections between the cardboard boundary and the background workpiece, the Canny operator extracts image-gradient edges, which are then dilated, as shown in Step 3 of Figure 12. The dilated edges form an exclusion band between the target and the background. This band is removed from the initial candidate mask to break weak pixel connections between the cardboard region and the background, thereby preventing undesired merging of the target and background during subsequent connected-component analysis.

To further improve the mask quality, morphological opening is applied to the above result. The opening operation can effectively remove small isolated noise caused by factors such as surface reflection and dust, while eliminating the residual thin connections between the cardboard contour and adjacent mixed-color regions, improving the completeness of the target region and the independence of the connected components, and providing a more stable input for subsequent geometric feature analysis.

Because non-target connected components that satisfy the color condition may remain after color thresholding and morphological processing, the method further applies multi-parameter filtering based on geometric attributes. Using the physical dimensions of the cardboard, the area, aspect ratio, and rectangularity of each connected component are evaluated against the constraints listed in Table 1. Components that do not satisfy the target characteristics are removed. The resulting 2D cardboard mask, shown in Step 5 of Figure 12, provides a reliable basis for subsequent depth-map mapping and 3D point-cloud extraction.

The color thresholds of the primary branch and the geometric acceptance criteria of both branches were calibrated for the two fixed production stations and the white and brown interlayer cardboard materials used in the experiments. The color-based branch is therefore intended for the calibrated camera pose, illumination configuration, station ROI, and cardboard appearance. Changes in illumination, camera exposure, or cardboard color may shift the observed color distribution and alter the connected-component structure. Similarly, transferring the procedure to another station may invalidate the station ROI, reference-plane correction, and station-specific geometric constraints.

A recalibration procedure is required before deployment under changed conditions. First, representative RGB-D samples are acquired for the cardboard, heat exchanger, background, and empty-station states under the intended production illumination. The station ROI is then updated, and the pallet reference plane is reacquired if the camera pose has changed. Next, the color thresholds are adjusted using the observed cardboard and background distributions, while the area, aspect-ratio, and rectangularity limits in Table 1 are revalidated using manually labelled calibration samples. The RANSAC threshold schedule and the geometric acceptance tolerances of the fallback branch are then checked against the expected cardboard dimensions, component count, and inter-component height consistency. The calibrated parameters are finally verified on independent hold-out scenes before robot motion is enabled.

During operation, a color-based result is accepted only when it satisfies both the geometric constraints and the expected component-count condition. If these conditions are not met, the geometry-based fallback is activated.

In the fallback branch, dominant planes are successively extracted using RANSAC with distance thresholds of 5, 10, 15, and 20 mm, and the uppermost plane is selected according to its mean depth under the adopted camera-coordinate convention. Euclidean clustering is then performed on the selected plane. For each cluster, a local planar coordinate system is established by principal-component analysis, and its robust projected dimensions are calculated from the 1st and 99th percentiles along the two principal directions. A candidate is accepted only when its projected long side, short side, and area satisfy the predefined cardboard geometry, when the candidate group contains the expected two or three components, and when these components exhibit consistent dimensions, area, and mean height. The thresholds are examined in ascending order, and a geometrically valid result with an inconsistent component count is not accepted; instead, the search continues at the next threshold. If two adjacent cardboard regions form a single cluster, the projected points are discretized into an occupancy grid, and the cluster is divided at the central low-occupancy valley before the same geometric constraints are reapplied. If no valid candidate group is obtained at any RANSAC threshold, the surface is not classified as cardboard and is passed to the non-cardboard processing branch.

**Figure 13:**
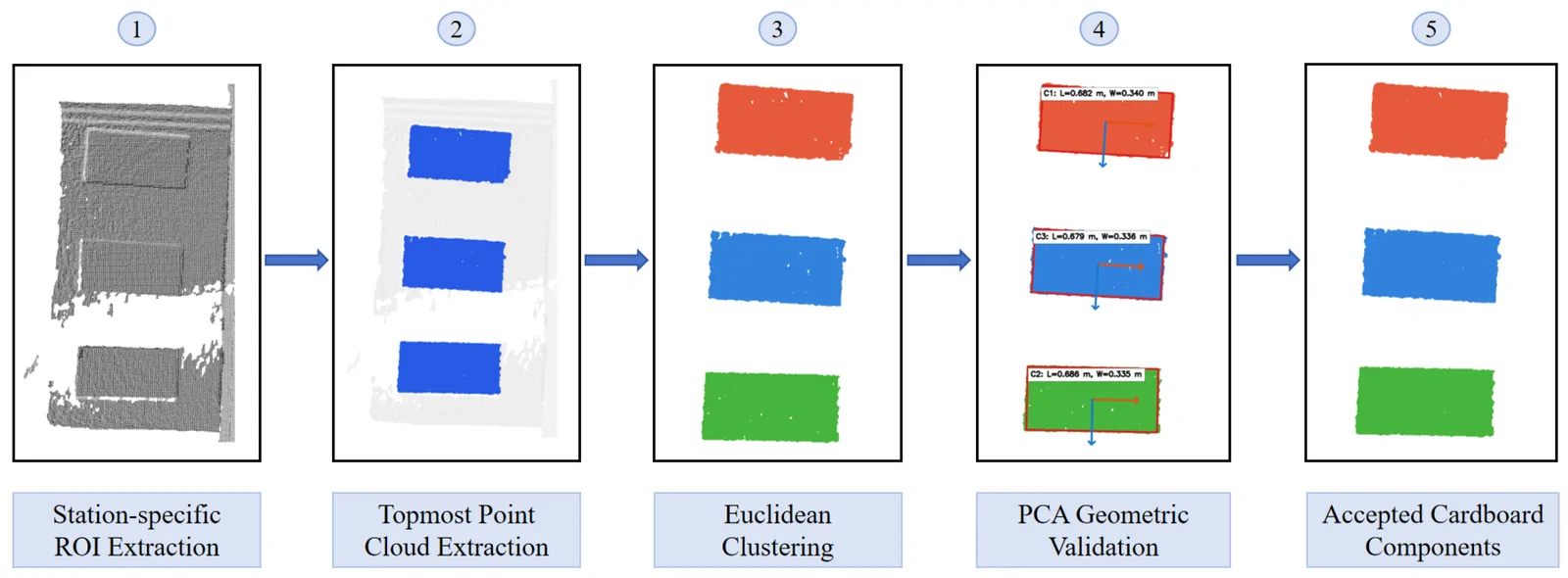
Processing flow of the geometry-based fallback for interlayer-cardboard recognition: the arrows and numbers indicate the sequence of the algorithm steps.

For a cluster projected onto the local planar axes *u* and *v*, the robust side lengths are defined as

(5) $$d_{u}=Q_{0.99}\left(u\right)-Q_{0.01}\left(u\right),\;d_{v}=Q_{0.99}\left(v\right)-Q_{0.01}\left(v\right)$$

where $Q_{p}$ denotes the *p*-quantile. The long side, short side, and projected area are then $L=\max(d_{u},d_{v})$, $W=\min(d_{u},d_{v})$, and A=LW, respectively. These quantities are compared with the predefined cardboard dimensions and tolerances. The complete processing sequence of the geometry-based fallback is summarized in Figure 13.

**Figure 14:**
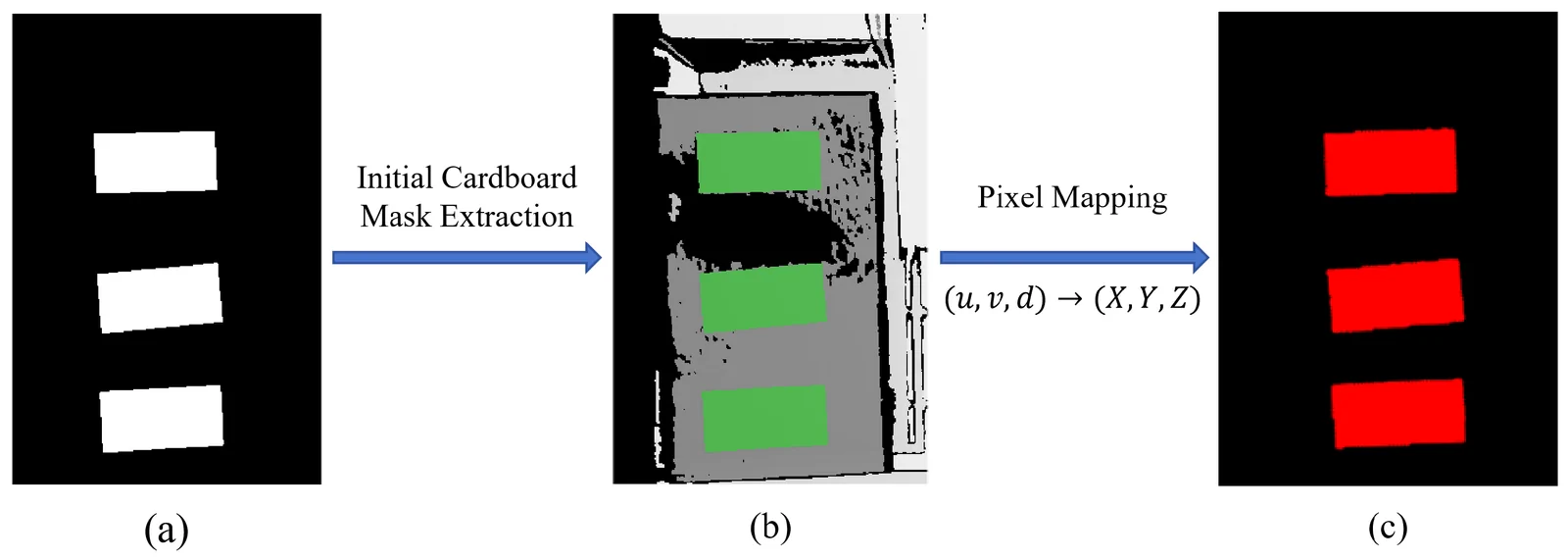
Cardboard point-cloud extraction through spatial mapping: (**a**) 2D cardboard mask; (**b**) target cardboard depth region; (**c**) cardboard point cloud.

The two branches produce different intermediate outputs. The geometry-based fallback directly returns the accepted three-dimensional cardboard components, whereas the color-based branch provides a two-dimensional cardboard mask. For the latter, the correspondence between the mask pixels and the three-dimensional points is established using the spatially aligned color and depth images through mask-constrained back-projection, as shown in Figure 14.

Let D(u,v) denote the aligned depth image. The system uses the binary mask of the target cardboard produced by the 2D image-processing module to isolate the corresponding depth pixels. As shown in Figure 14a, the 2D cardboard mask is denoted by $M\left(u,v\right)\in \left\{0,1\right\}$ and is directly applied to the aligned depth image. As shown in Figure 14b, the system traverses only pixels within the valid connected component(s) of the mask (i.e., M(u,v)=1) and extracts their corresponding depth values *Z*. Invalid or anomalous depth measurements caused by sensor noise or reflection are removed, and only candidate pixels within the effective working range are retained.

For the filtered valid pixels (u,v), the system maps them back to 3D space using the inverse transformation model of perspective projection. Let the focal lengths of the color camera along the corresponding axes be $f_{x}$ and $f_{y}$, and the principal point coordinates be $c_{x}$ and $c_{y}$; then the 3D spatial coordinate of the point in the color camera coordinate system, $P_{c}={\left[X_{c},Y_{c},Z_{c}\right]}^{T}$, can be reconstructed by the following equations:

(6) $$X_{c}=\frac{(u-c_{x})\cdot Z}{f_{x}}$$

(7) $$Y_{c}=\frac{(v-c_{y})\cdot Z}{f_{y}}$$

(8) $$Z_{c}=Z$$

To ensure the consistency of the coordinate reference in subsequent grasping tasks, the reconstructed point cloud is transformed from the color camera coordinate system back to the depth coordinate system. Let the corresponding inverse transformation rotation matrix be $R_{c\to d}$ and the translation vector be $T_{c\to d}$; the 3D point set of the cardboard $P_{out}$ is obtained through the homogeneous coordinate transformation:

(9) $$\left[\begin{array}{c} P_{out}\\ 1 \end{array}\right]=\left[\begin{array}{cc} R_{c\to d} & T_{c\to d}\\ 0 & 1 \end{array}\right]\left[\begin{array}{c} P_{c}\\ 1 \end{array}\right]$$

For the color-based branch, the mapping and reconstruction shown in Figure 14c isolate the target cardboard and provide its 3D point cloud for subsequent pose estimation.

#### 2.2.3. Heat Exchanger Point Cloud Extraction and Precise Registration

After station extraction and point-cloud preprocessing, the point cloud contains surface points from the target heat exchanger and residual non-target points. RANSAC and Euclidean clustering can then be used to segment the heat-exchanger surface point cloud using appropriate threshold parameters.

**Figure 15:**
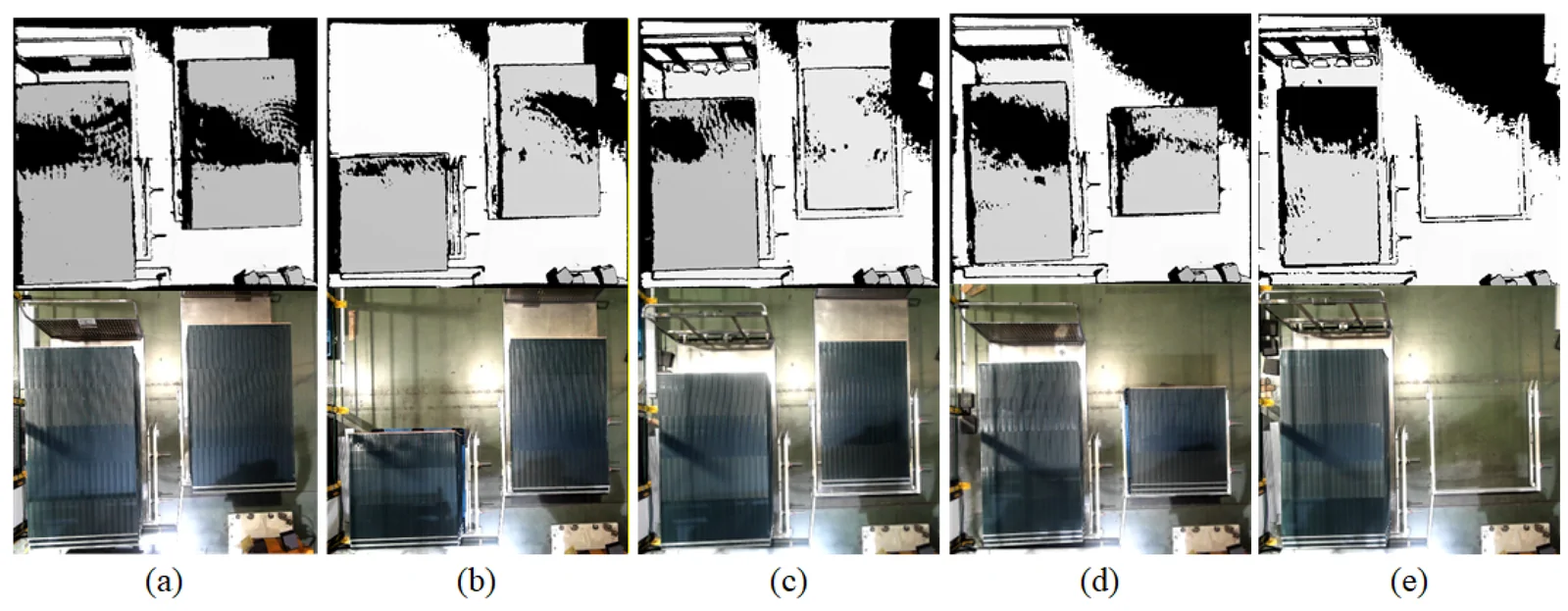
Missing regions in the depth image: (**a**–**e**) representative samples; the first row shows depth images, where the black regions indicate missing depth information, and the second row shows the corresponding color images.

After extraction, the incomplete heat-exchanger scan is registered with the corresponding CAD template using a model-specific three-stage procedure. The procedure combines projected-area yaw alignment, tail-constrained longitudinal anchoring, and inverse CAD-template mapping. It is designed for known heat-exchanger models whose global dimensions and asymmetric tail geometry are available as prior information. Accordingly, the intended application is fixed-station localization of known heat-exchanger models within a calibrated workspace. The reflection-induced missing regions are illustrated in Figure 15.

**Figure 16:**
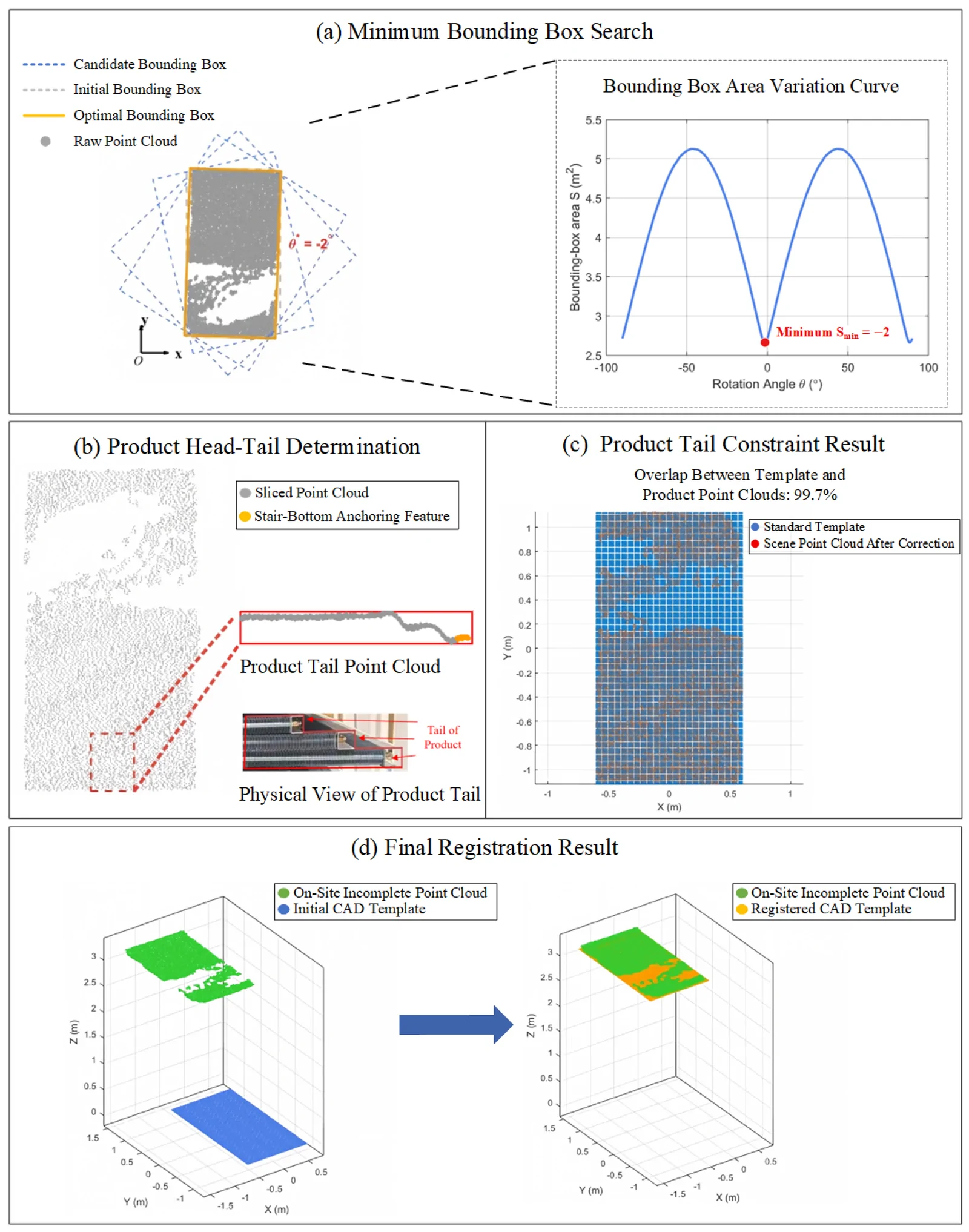
Point-cloud registration pipeline based on projection extrema and feature anchoring: the arrow in (**d**) indicates the transition from before registration to after registration.

**Figure 17:**
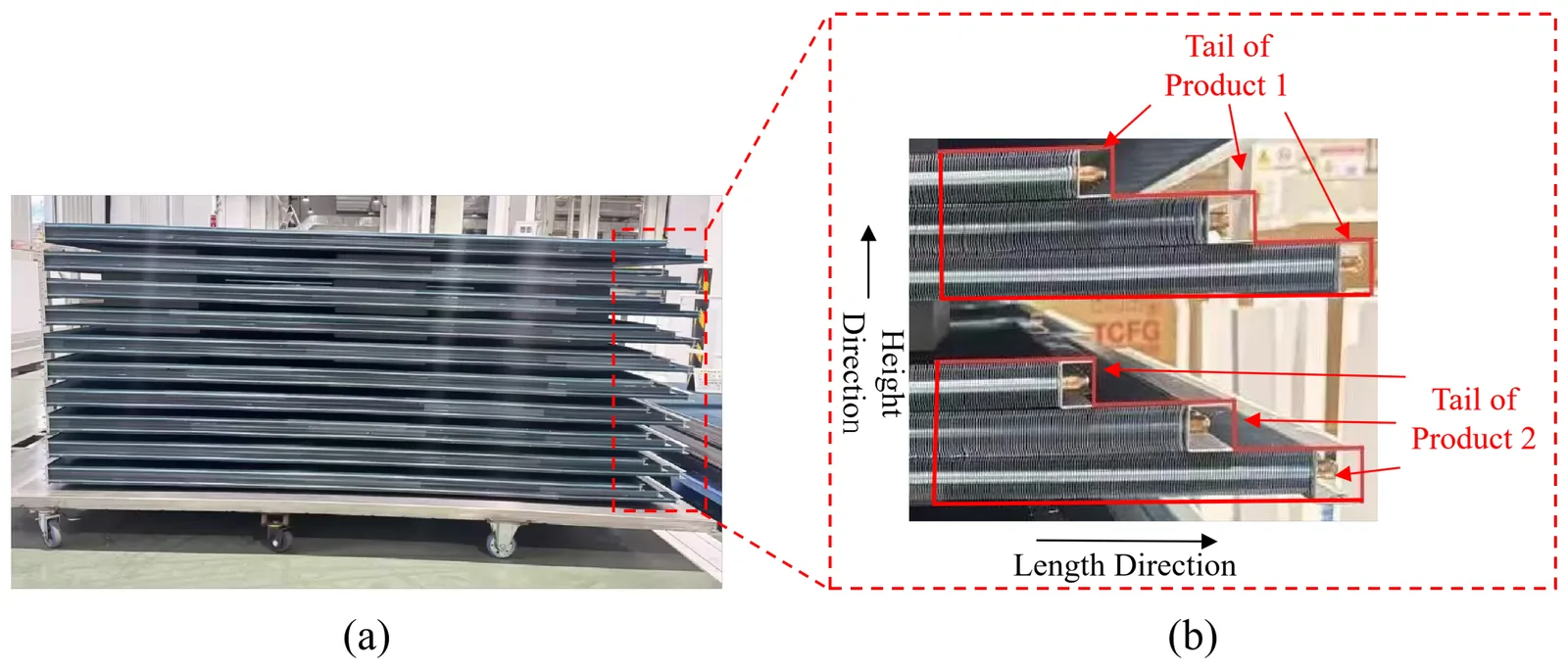
Heat exchanger and its tail: (**a**) heat exchanger; (**b**) tail of the heat exchanger.

**Figure 18:**
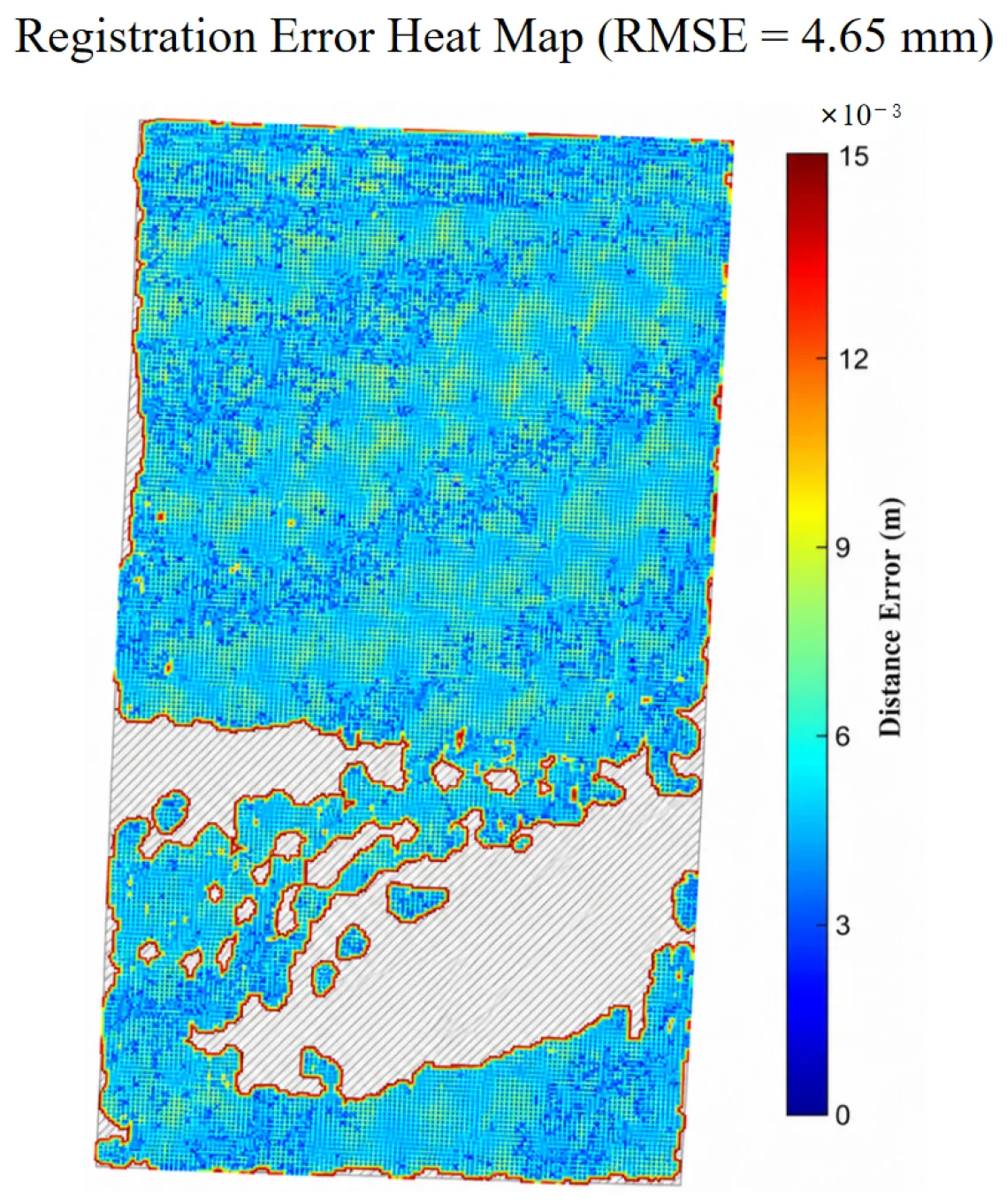
Distance-error heat map of the product point cloud and template point cloud after registration. The hatched region denotes a missing region in the raw point cloud that is reconstructed but excluded from the error statistics.

1. Yaw-angle estimation and lateral alignment based on bounding-box extrema. After plane extraction and normal-vector correction, the principal plane of the product point cloud is approximately parallel to the XOY plane of the camera coordinate system. Let *a* and *b* denote the true length and width of the product, respectively. Let θ denote the yaw angle of the point cloud about the Z-axis, and let $L_{x}$ and $L_{y}$ denote the theoretical projected lengths on the X- and Y-axes, respectively. These lengths correspond to the width and length of the bounding box. According to the geometry of 2D rotational projection, the projected bounding-box area *S* is given by:
  (10) $$L_{x}=a\left|\cos\theta \right|+b\left|\sin\theta \right|$$
  (11) $$L_{y}=a\left|\sin\theta \right|+b\left|\cos\theta \right|$$
  (12) $$S=L_{x}L_{y}=ab+\frac{1}{2}\left(a^{2}+b^{2}\right)\left|\sin(2\theta )\right|$$
  Theoretical analysis shows that the projected bounding-box area *S* reaches its minimum when the rotation angle θ approaches kπ/2(k∈Z). The right-angle boundaries of the projected point cloud are then parallel to the X- and Y-axes. Accordingly, the algorithm performs an exhaustive search for the rotation angle that minimizes the area; the search process and optimization curve are shown in Figure 16a. After the initial yaw angle has been obtained, a long-side/short-side ambiguity may remain because of substantial incompleteness. Because the lower short-side boundary is relatively well preserved in the incomplete product point cloud, a physical-dimension prior is used. Specifically, the algorithm compares the projected lengths $L_{x}$ and $L_{y}$ at the minimum area with the true short-side dimension *b* of the product. If $L_{y}$ is closer to *b*, the short side is considered to be aligned with the Y-axis. An additional 90° rotation is then applied to align the short side of the product with the X-axis. This joint estimation based on the global projection extremum and local-prior verification determines the absolute yaw orientation of the original incomplete point cloud about the Z-axis and completes its axial alignment. The geometric center of the short side at the minimum projected width is then calculated to obtain the short-side centerline of the product. Because the coordinate origin of the standard template point cloud is located at its geometric center, translation compensation is applied to the product point cloud at the optimal rotation angle. The point cloud is translated along the X-axis until its short-side centerline coincides with the Y-axis of the coordinate system. The lateral degrees of freedom of the product point cloud and template point cloud are then aligned.
2. Longitudinal anchoring based on the stepped tail geometry. After lateral alignment, the point cloud still has an unknown longitudinal translational offset. Because high-reflectivity-induced missingness is spatially nonuniform, the centroid of the incomplete observation can deviate substantially from that of the complete product, making global centroid alignment unreliable for longitudinal localization. The stepped tail geometry is therefore introduced as a model-specific structural reference for longitudinal anchoring. As shown in Figure 17, the heat exchanger comprises multiple columns of single-layer modules with unequal lengths, and its tail has a pronounced stepped geometric structure. The tail region is selected as the anchoring area based on the observation that its local geometric structure is relatively stable under normal acquisition conditions. This local depth-gradient feature is used to determine the head–tail orientation and anchor the product point cloud. Specifically, the bounding-box regions at the two longitudinal ends of the current product point cloud, each accounting for 15% of its length along the Y-axis, are extracted as feature-detection slices. Because industrial 3D cameras are prone to edge-related flying-point outliers, the algorithm calculates, for the end-slice point-cloud sets $P_{\mathrm{top}}$ and $P_{\mathrm{tail}}$, the difference between the 98th and 2nd percentiles along the depth (Z-axis) direction. This yields the depth ranges $\Delta Z_{\mathrm{top}}$ and $\Delta Z_{\mathrm{tail}}$ at the head and tail ends. These ranges are compared with the depth-gradient threshold $T_{d}$ determined by the number of product columns. When $\Delta Z>T_{d}$ for an end slice, the region is considered to exhibit a pronounced stepped feature and is identified as the product tail, as shown in Figure 16b. To improve robustness to severe noise, if both end ranges satisfy the threshold or neither does, the system uses an asymmetric centroid-shift strategy to determine the head–tail orientation. Because the incomplete product has a highly asymmetric spatial distribution, with substantial missing data in its upper part and a relatively complete lower part, the true 3D centroid of the target point cloud tends to shift toward the tail relative to the center of its geometric bounding box under the evaluated acquisition geometry. The system calculates the longitudinal centroid $Y_{\mathrm{centroid}}$ of the point cloud and the bounding-box center $Y_{\mathrm{center}}$, then treats the side toward which the centroid is biased as the physical tail. If the true tail lies in the positive half-axis region of the current bounding box, the point-cloud orientation is opposite to the predefined standard pose. The system then constructs a 180° rigid rotation matrix about the local transverse axis and applies it to the target point cloud. Once the absolute spatial orientation of the product point cloud has been determined, the stepped edge is used as a high-confidence spatial reference. The longitudinal extreme coordinate of the tail, $Y_{\mathrm{tail}\_\mathrm{edge}}$, is extracted from the corrected point cloud to construct a 3D translation constraint vector. The observed incomplete product point cloud is then rigidly translated toward the ideal CAD template until their tails are aligned in space.
3. Inverse mapping of the template point cloud. Following the preceding steps, the projected-area overlap ratio between the incomplete product point cloud and the template point cloud is 99.7%, and the two exhibit a high degree of pose consistency in the reference coordinate system, as shown in Figure 16c. Let $T_{f}$ denote the overall transformation matrix that maps the incomplete product point cloud from the original space to the aligned state. By the reversibility of rigid-body transformations, the inverse transformation matrix $T_{f}^{-1}$ is obtained and directly applied to the template point cloud:
  (13) $$P_{template}^{original}=T_{f}^{-1}\cdot P_{template}^{aligned}$$
  This inverse mapping transforms the high-fidelity template point cloud to the observed spatial pose of the original incomplete point cloud, as shown in Figure 16d. The transformed template point cloud then replaces the original incomplete point cloud and provides complete data for subsequent high-precision grasping-pose estimation. Figure 18 shows the distance-error heat map between the reconstructed point cloud output by the proposed algorithm and the reference CAD template. The distance map indicates small residual errors in the evaluated region and shows that the registration mechanism provides a geometrically complete point cloud for calculating the final spatial coordinates of the heat exchanger.

#### 2.2.4. Grasping Pose Estimation

After the interlayer-cardboard point cloud has been obtained through either mask-constrained back-projection or the geometry-based fallback, and after inverse registration and template reconstruction of the heat-exchanger point cloud, the target point clouds have estimated spatial poses. The final task of the vision system is to estimate the grasping the pose of the target workpiece (the heat exchanger or interlayer cardboard) and convert it to motion commands in the robot base coordinate system using the hand–eye calibration system.

Because the point-cloud data supplied to the pose estimator have high completeness and an approximately uniform spatial distribution, the grasp point can be located by calculating the geometric centroid of the global point cloud. Let the valid point-cloud set of the target obtained through the preceding process be $P={\left\{p_{i}\left(x_{i},y_{i},z_{i}\right)\right\}}_{i=1}^{N}$; then the geometric center coordinates of the target workpiece, $P_{c}={\left(X_{c},Y_{c},Z_{c}\right)}^{T}$, can be obtained as follows:

(14) $$X_{c}=\frac{1}{N}\sum_{i=1}^{N}x_{i},\;Y_{c}=\frac{1}{N}\sum_{i=1}^{N}y_{i},\;Z_{c}=\frac{1}{N}\sum_{i=1}^{N}z_{i}$$

Owing to the introduction of the high-fidelity template point cloud and the strict mask filtering, the influence of local reflection-induced missing data on centroid calculation is reduced, improving the precision of spatial localization.

The rotation angle in the grasping pose determines the degree of yaw alignment between the end effector (gripper or suction cup) and the geometric principal axis of the workpiece. Since plane correction of the reference plane has been completed in the preprocessing stage, and the principal plane of the workpiece is strictly parallel to the XOY plane of the camera coordinate system, only the in-plane rotation (the yaw angle $R_{z}$ around the Z-axis) needs to be considered in the pose estimation here.

To estimate the rotation angle $R_{z}$ about the Z-axis, the oriented bounding box (OBB) algorithm is used to extract the geometric principal axis of the target point cloud on the XOY projection plane. Let the extracted major-axis direction vector be $V_{major}={\left(v_{x},v_{y}\right)}^{T}$; the rotation angle about the Z-axis is then obtained using the arctangent function:

(15) $$R_{z}=\arctan\left(v_{x},v_{y}\right)$$

Using the estimated parameters, the vision system generates the grasping-pose vector $P_{grasp}={\left[X,Y,Z,R_{z}\right]}^{T}$ for the industrial robot. Together with the pre-calibrated hand–eye transformation matrix, this vector is mapped to the robot base coordinate system and converted into executable kinematic commands, completing the vision-guided grasping process. The required translational accuracy of this pose follows the clearance-based limits derived in Section 2.1, while the yaw estimate is additionally constrained by the lateral clearance consumed by the end displacement of the product.

## 3. Experiments and Analysis

### 3.1. Experimental Setup

The experimental system comprises a 3D camera, an industrial computer, a programmable logic controller (PLC), and associated mechanical actuators. The algorithm is implemented in C++ using RobotVisionSuite and executed on an industrial computer equipped with an i5-7500 processor and 16 GB of DDR memory. Each interlayer cardboard measures 700 mm × 350 mm, with a nominal thickness of 30 mm and a measured thickness of typically 25–28 mm under production conditions. Table 2 lists the dimensions of the main product models used in the experiments. The sample sets used for each evaluation are described in the corresponding subsections.

All experiments were conducted with the cameras mounted at their installed production positions, while the normal production-line illumination and camera exposure settings were kept fixed. The evaluated variations, therefore, arose from product model, stacking configuration, stack height, and naturally occurring or artificially controlled point-cloud missingness. Camera pose and illumination were not systematically varied. Unless otherwise stated, the reported robustness consequently refers to these factors within the calibrated station configurations.

Table 3 summarizes the principal parameters and distinguishes those that are fixed across product models from those that require model-, station-, or material-specific calibration. It thereby clarifies the extent to which the method can be transferred to another production line without retuning.

### 3.2. Plane Correction Performance and Repeatability

To evaluate plane-correction performance over the working-height range of the production line, ten planar point-cloud samples were obtained from ten consecutive layers of the same material cart. The median surface depths of these samples ranged from approximately 3.17 to 4.02 m. An independently acquired pallet-plane point cloud was used as the common correction reference. The correction transformation estimated from this reference plane was kept unchanged and applied to all ten product-plane samples.

The uppermost planar region of each heat exchanger was extracted to avoid the inherent depth steps of the multicolumn product structure. Two metrics were used for quantitative evaluation. First, the robust Z-direction depth span was defined as $\Delta Z_{\mathrm{robust}}=P_{99}\left(Z\right)-P_{1}\left(Z\right)$, where $P_{1}$ and $P_{99}$ denote the 1st and 99th percentiles of the Z coordinates, respectively. This metric reduces the influence of isolated flying points. Second, the plane inclination was calculated as the angle between the fitted plane normal and the camera’s Z-axis. The point-to-plane RMSE of the RANSAC inliers was additionally reported as an empirical measure of within-plane measurement dispersion.

**Figure 19:**
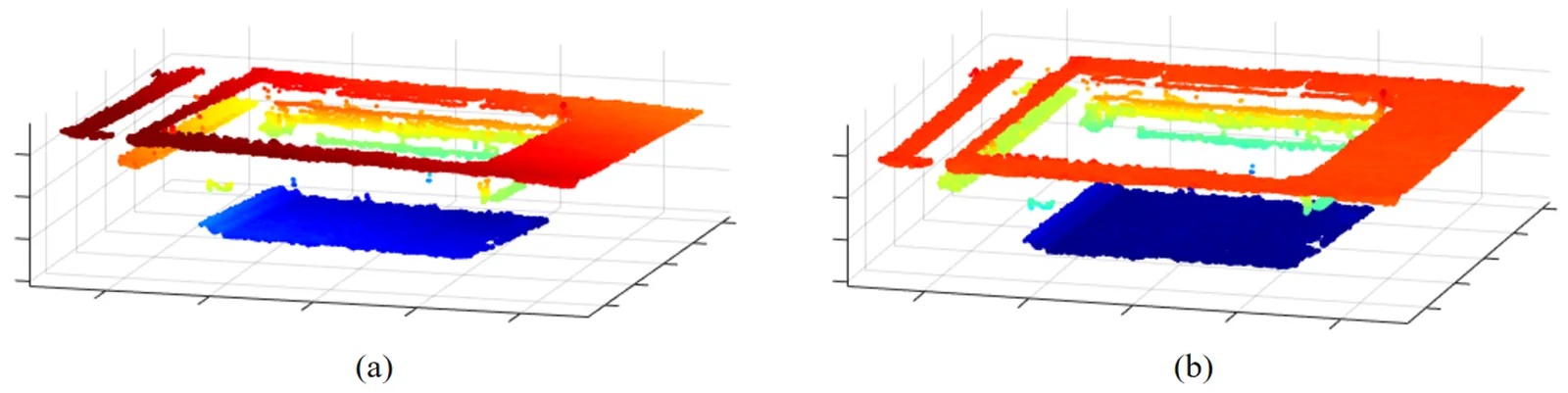
Representative product-plane point cloud: (**a**) before plane correction; (**b**) after plane correction.

Figure 19 presents a representative point-cloud sample before and after plane correction. Before correction, the fitted product plane exhibits a visible inclination relative to the XOY plane because of the installed camera pose. After the common correction transformation is applied, the product plane becomes approximately parallel to the XOY plane, providing a consistent coordinate reference at different stack heights.

As shown in Table 4, the mean robust Z-direction depth span decreased from 54.11±2.32 mm before correction to 15.54±1.39 mm after correction, corresponding to a mean reduction of 38.57 mm, or 71.29%. The 95% confidence interval of the paired mean reduction was 37.52–39.62 mm. The mean plane inclination decreased from 2.234±0.122° before correction to 0.241±0.122° after correction, corresponding to an 89.22% reduction. The 95% confidence interval of the inclination reduction was 1.982–2.005°. Paired Wilcoxon signed-rank tests showed significant reductions in both the robust Z-direction span and the plane inclination (p=0.00195 for both metrics).

The point-to-plane RMSE of the ten extracted planar regions was 2.79±0.35 mm. This value characterizes the within-plane dispersion caused by surface variation and depth-measurement noise rather than the magnitude of the rotational correction. Its magnitude is consistent with the nominal measurement accuracy of 3.4 mm was reported for the camera at a working distance of 4.3 m.

The repeatability of the reference-plane estimation was further evaluated through 100 independent RANSAC runs using different random seeds. The fitted reference-plane tilt was 2.049±0.042°. The angular deviation of the fitted normals from their mean direction was 0.0456±0.0392°, with a maximum deviation of 0.1591°. Across these runs, the mean post-correction inclination of the ten layers was 0.209±0.037°, and the mean corrected robust Z-direction span was 15.13±0.46 mm. These results demonstrate that the RANSAC-based reference-plane estimation and the resulting correction remain stable over repeated executions.

### 3.3. Interlayer Cardboard Point Cloud Extraction: Experimental Results and Analysis

To quantitatively evaluate the color-based primary branch and the geometry-based fallback under the calibrated conditions of the two workstations, this section uses 129 paired RGB and point-cloud samples, comprising 62 samples from Station 1 and 67 samples from Station 2. The Intersection over Union (IoU) between the binary mask and a manually annotated reference mask is used to measure the overlap between the cardboard mask extracted by the color-based branch and the physical boundary of the cardboard. It is calculated as follows:

(16) $$IoU=\frac{\mathrm{Area}\left(M_{\mathrm{predict}}\cap M_{\mathrm{gt}}\right)}{\mathrm{Area}\left(M_{\mathrm{predict}}\cup M_{\mathrm{gt}}\right)}$$

where $M_{\mathrm{predict}}$ is the 2D cardboard mask output by the algorithm, and $M_{\mathrm{gt}}$ is the manually annotated reference mask of the cardboard. A color-based result is regarded as correct only when its component count agrees with the expected count supplied by the production-task configuration and its IoU is not lower than 0.80. The manually annotated mask is used only for offline evaluation and does not participate in online branch selection.

**Figure 20:**
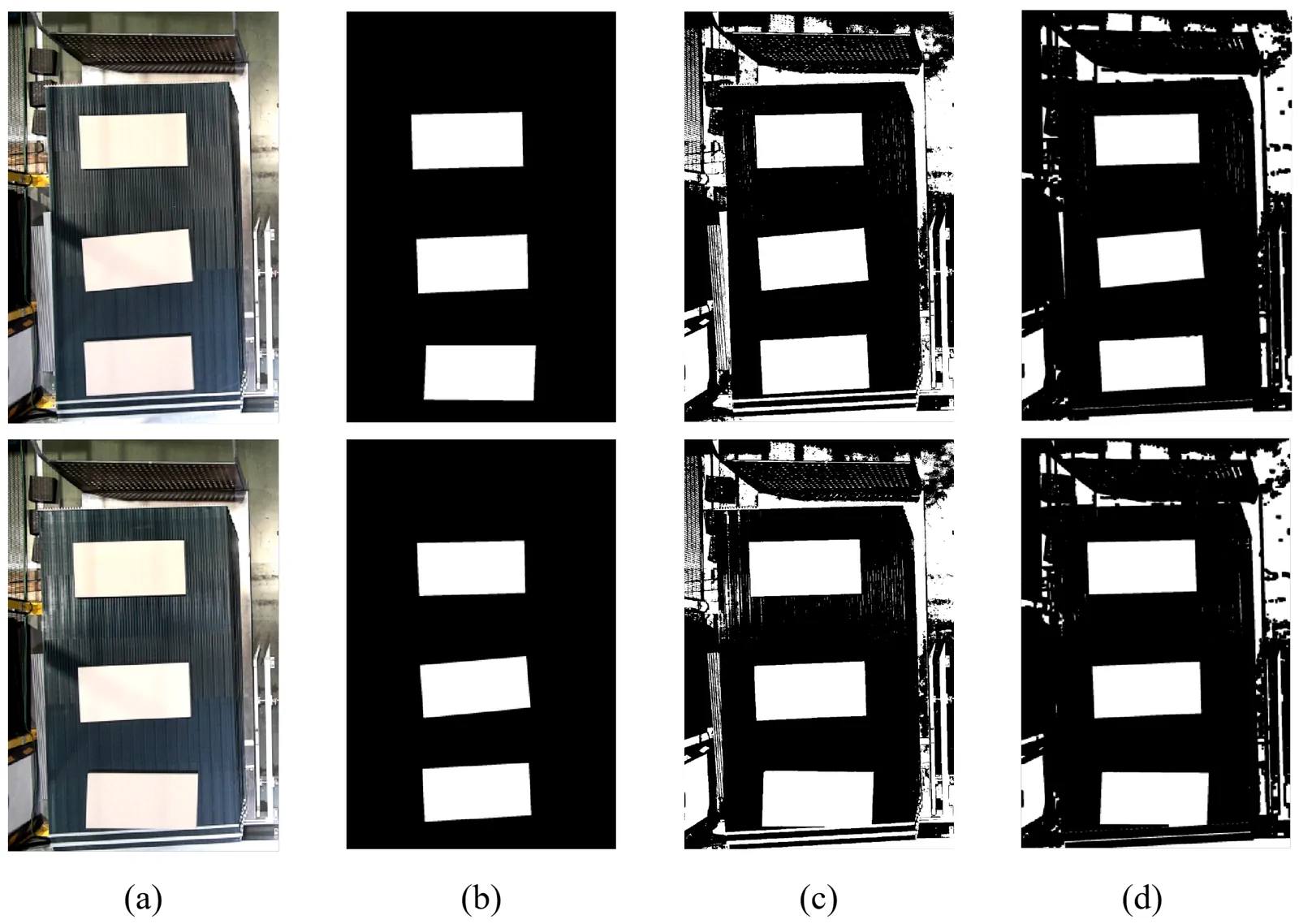
Comparison of interlayer-cardboard extraction using morphological processing: (**a**) raw ROI image; (**b**) manually annotated reference mask; (**c**) basic color-threshold segmentation mask; (**d**) mask after the proposed morphological processing.

**Figure 21:**
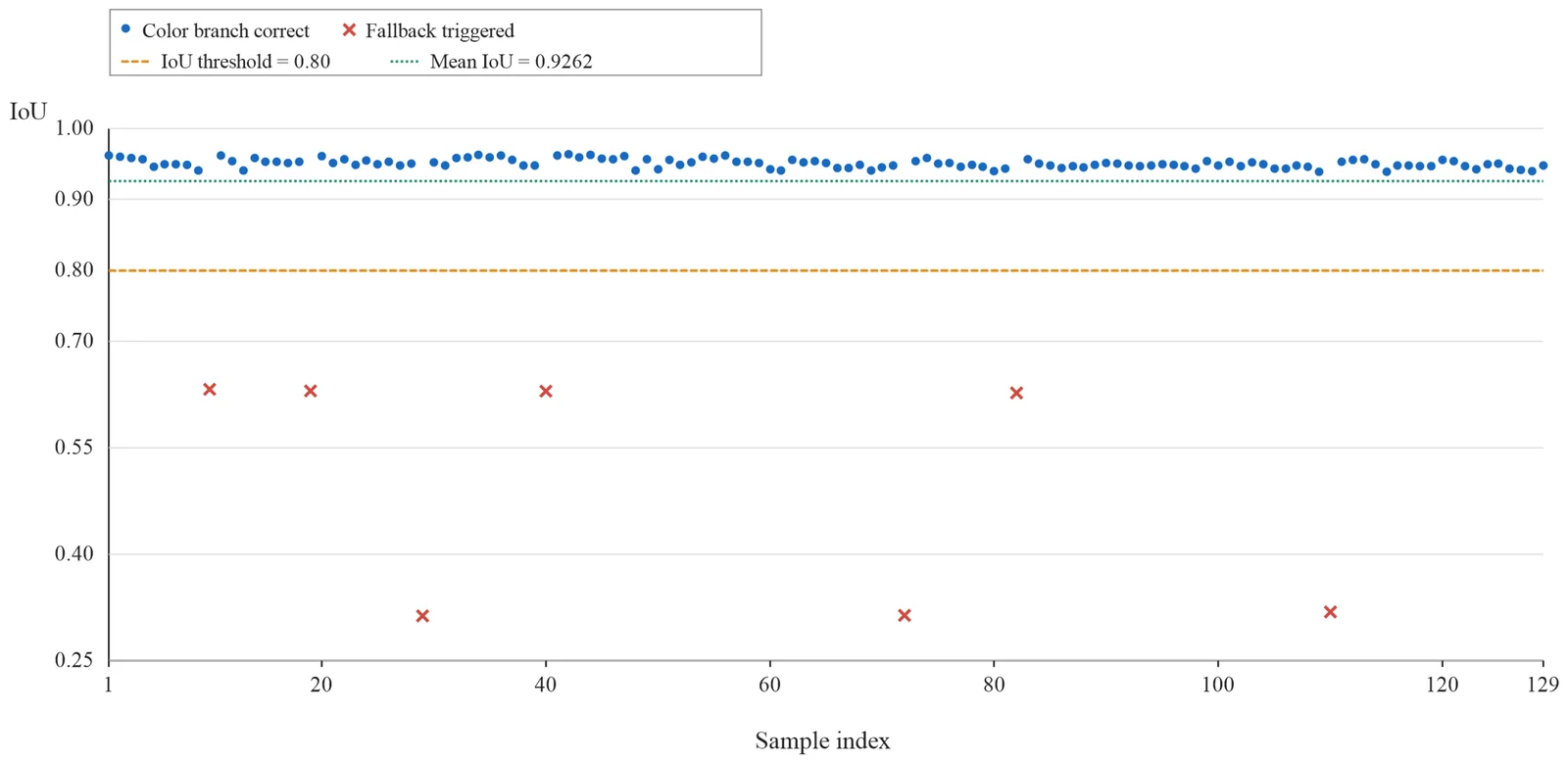
IoU distribution of the color-based primary branch over the evaluated samples.

**Figure 22:**
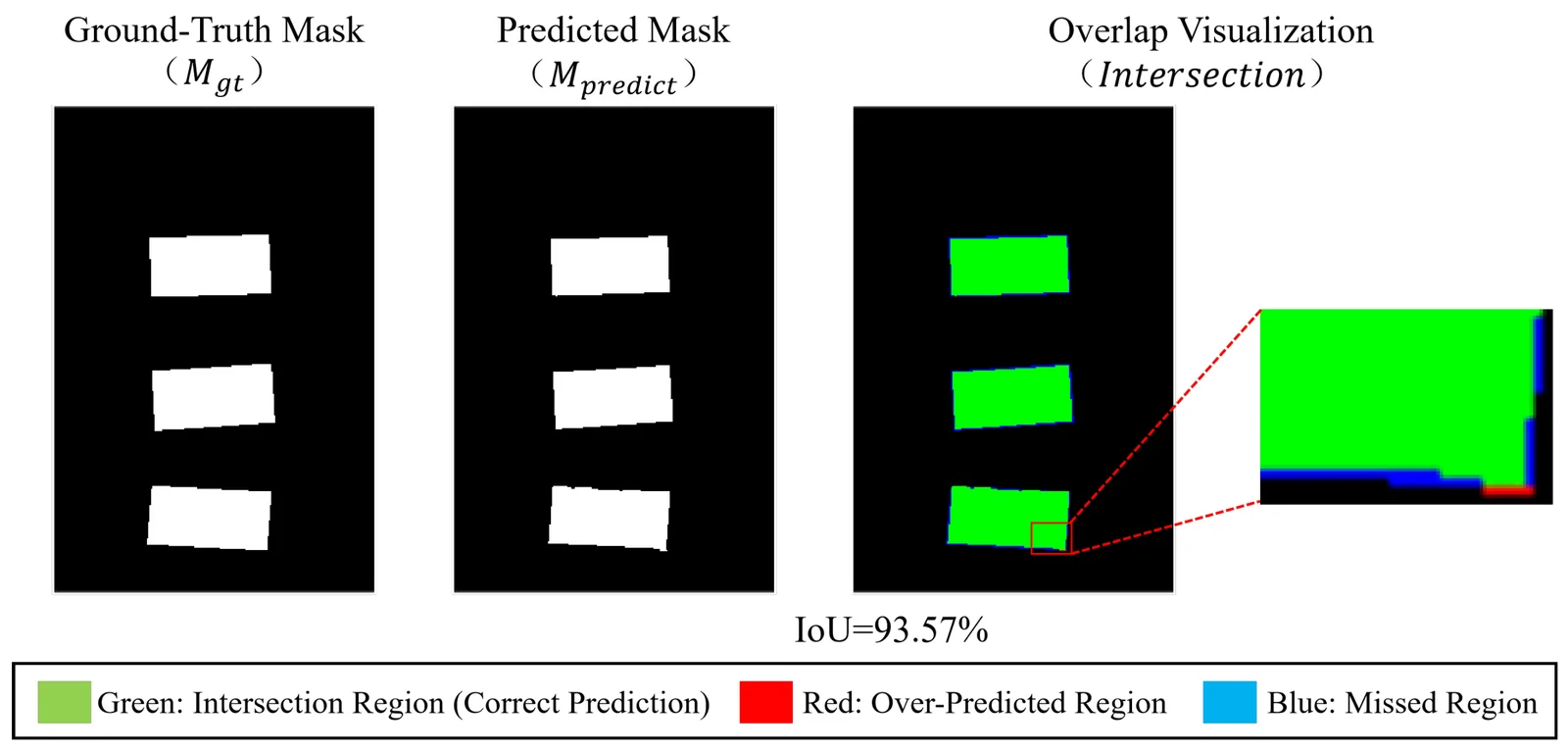
Comparison of cardboard masks for representative samples: green indicates the intersection region (correct prediction), red indicates the over-predicted region, and blue indicates the missed region.

The proposed morphological preprocessing strategy is first compared with single-color threshold segmentation. As shown in Figure 20c, basic threshold segmentation is prone to misclassification when the cardboard edges and the underlying product have similar colors or overlapping shadows. By incorporating Canny edge detection and morphological opening, the proposed algorithm demonstrates improved mask completeness in the presence of interference. As shown in Figure 20d, it removes weak connections between the target and mixed-color background while repairing internal defects. The sample-wise IoU distribution is shown in Figure 21, and representative cardboard-mask results are shown in Figure 22. The mean IoU over all 129 samples was 92.62%. Among the 122 samples that satisfied both the component-count and IoU criteria, the mean IoU was 95.09%, corresponding to a color-branch recognition accuracy of 94.57%. These results show that the color-based branch correctly processed most samples under the calibrated illumination and cardboard conditions, while the remaining cases required the geometry-based fallback.

As shown in Table 5, seven samples were transferred to the fallback branch. In three cases, the color-based branch produced only one valid component; in the other four, the detected component count was inconsistent with the expected count. The fallback correctly recovered all seven cases by applying sequential plane extraction, clustering, and geometric validation to the point cloud. IoU is reported only for the two-dimensional color-based branch, whereas fallback success is determined by whether the expected number of geometrically valid three-dimensional cardboard components is returned.

### 3.4. Prior Template Registration Performance: Experimental Results and Analysis

#### 3.4.1. Overall Registration Performance

To comprehensively evaluate algorithm performance, 44 sets of raw heat-exchanger point-cloud data were collected from the production line. Each set contains approximately 8–12 samples, yielding a total of 436 samples with different stacking poses. The samples cover five product models, including two- and three-column variants, and are therefore reported both by missing-data condition and by product model below. To quantify the degree of incompleteness of the product point cloud, the system calculates the proportion of invalid depth pixels $R_{miss}$ within the extracted region of interest (ROI) of the heat-exchanger depth image as

(17) $$R_{miss}=\frac{N_{invalid}}{N_{total}}\times 100\%$$

**Figure 23:**
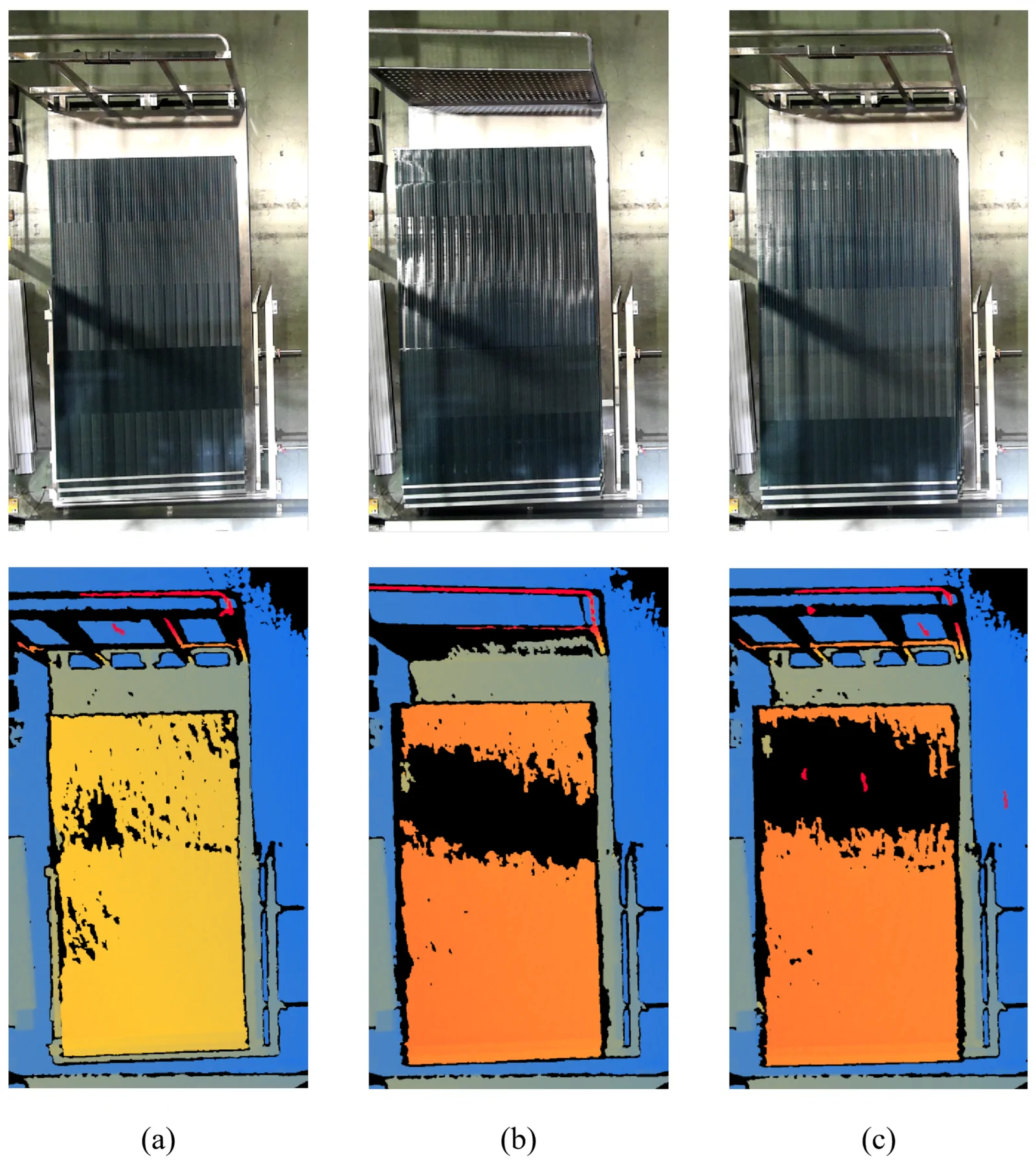
Representative samples under different missing-data conditions: (**a**) low; (**b**) moderate; (**c**) severe.

According to the calculated heat-exchanger point-cloud missing rate, the experimental samples are divided into three missing-data conditions: low $\left(R_{miss}<15\%\right)$, moderate $\left(15\%\leq R_{miss}\leq 30\%\right)$, and severe $\left(R_{miss}>30\%\right)$. The dataset contains 193 samples with low missing-data rates, 199 with moderate rates, and 44 with severe rates. Figure 23 shows representative RGB images for the three conditions together with their corresponding depth images.

For each sample, a manually annotated reference pose was obtained by interactively aligning the corresponding complete CAD-template point cloud with the observable geometric regions of the product point cloud. The resulting transformed CAD template was used as the reference template in the following error calculations.

Let ${\hat{c}}_{i}$ and $c_{i}^{\mathrm{ref}}$ denote the centroids of the registered template and the corresponding reference template for sample *i*, respectively. The translation error $e_{T,i}$ and nearest-neighbor RMSE $e_{\mathrm{RMSE},i}$ of sample *i* are defined separately as

(18) $$e_{T,i}={∥{\hat{c}}_{i}-c_{i}^{\mathrm{ref}}∥}_{2}$$

(19) $$e_{\mathrm{RMSE},i}=\sqrt{\frac{1}{M_{i}}\sum_{k=1}^{M_{i}}\min_{q\in Q_{i}^{\mathrm{ref}}}{∥{\hat{p}}_{i,k}-q∥}_{2}^{2}}$$

where ${\hat{p}}_{i,k}$ is the *k*th point of the registered template retained by the evaluation mask, $M_{i}$ is the number of such points, and $Q_{i}^{\mathrm{ref}}$ is the reference-template point set. The evaluation mask excludes regions in which the raw product observation contains no valid depth data; therefore, template-mapped points in these unobserved regions are not included in the nearest-neighbor RMSE. For a group containing *N* samples, the reported mean translation error and mean nearest-neighbor RMSE are calculated separately as

(20) $${\bar{e}}_{T}=\frac{1}{N}\sum_{i=1}^{N}e_{T,i}$$

(21) $${\bar{e}}_{\mathrm{RMSE}}=\frac{1}{N}\sum_{i=1}^{N}e_{\mathrm{RMSE},i}$$

To characterize the typical error of the sample-wise error distribution, the median (P50) and the 95th percentile (P95) of the translation error were also calculated. The median is the value below which 50% of the sample-wise translation errors fall, whereas P95 is the value below which 95% of them fall. In addition, 95% confidence intervals (CIs) for the mean translation error and mean nearest-neighbor RMSE were estimated using 10,000 cluster-bootstrap resamples, with the 44 production sets treated as the resampling units to preserve the dependence among samples from the same set. Because the product’s longitudinal axis is geometrically equivalent under a 180° reversal, the yaw error was evaluated as the undirected long-axis angle. Let ${\hat{u}}_{i}$ and $u_{i}^{\mathrm{ref}}$ denote the unit longitudinal-axis vectors of the registered and reference templates, respectively. The sample-wise yaw error was calculated as

(22) $$e_{\psi ,i}=\arccos\;\left(\left|{\hat{u}}_{i}^{T}u_{i}^{\mathrm{ref}}\right|\right)$$

The registration-accuracy results are summarized in Table 6.

The difference between the median in Table 6 indicates that a small number of large errors increased the overall mean. The mean absolute X-, Y-, and Z-direction errors were 1.77 mm, 4.37 mm, and 0.48 mm, respectively, showing that the Y-direction error was the principal contributor to the translation error. Relative to the localization requirements defined in Section 2.1, two samples exceeded the X-direction tolerance, eight exceeded the Y-direction tolerance, and none exceeded the Z-direction tolerance. The two largest translation errors were 53.37 mm and 43.39 mm, both caused primarily by unusually large X-direction errors, and were retained in all aggregate statistics.

The aggregate results are affected not only by the missing-data level but also by differences in sample composition. In particular, the three missing-data groups contain unequal distributions of product models and stacking configurations. To examine the contribution of product-model composition, the registration results were further stratified by product model, as shown in Table 7.

The model-stratified results confirm that registration error varied with product model as well as missing-data level. Because the groups comprised independent production samples rather than paired, controlled degradations of the same samples, the observed non-monotonic variation reflects the combined effects of model distribution, stacking pose, and missing-region characteristics. The nearest-neighbor RMSE nevertheless remained within a relatively narrow range across the stratified groups.

In addition to registration accuracy, the computational cost of the proposed method was measured within the deployed RobotVisionSuite operator. The reported operator runtime covers the complete in-memory processing sequence from input point-cloud conversion, through the core registration procedure, to output point-cloud conversion. The same timing scope was applied to all 436 samples. The final implementation used a search interval of [−90°,90°], an angular resolution of 0.5°, and 361 candidate orientations. The mean operator runtimes for the low-, moderate-, and severe-missingness groups were 560.29 ms, 519.60 ms, and 454.38 ms per registration, respectively. Weighted by the corresponding sample counts of 193, 199, and 44, the overall mean operator runtime was 531.03 ms.

To identify the dominant computational stage, one representative sample was randomly selected from the low-missingness group. Its operator runtime was divided into input point-cloud conversion, application of the fixed reference-plane correction, projection-extrema search, head–tail determination and orientation, tail anchoring, template normalization, inverse template transformation, and output point-cloud conversion, as shown in Table 8. The correction stage applies the fixed rotation matrix obtained from the one-time reference-plane correction during system commissioning; it does not re-estimate the reference plane for each sample.

The projection-extrema search required 530.00 ms and accounted for 94.65% of the complete operator runtime because the projected coordinates were evaluated and sorted for each of the 361 candidate orientations. It was therefore identified as the principal computational bottleneck. By comparison, the input and output point-cloud conversions required only 1.55 ms and 2.75 ms, respectively. The complete operator runtime of this representative sample was 559.96 ms, close to the low-missingness group mean of 560.29 ms.

The input and output stages reported here refer to in-memory point-cloud conversion at the RobotVisionSuite operator interface. Disk-based file reading and writing, batch-data management, and result export were not included because they are not part of the deployed online operator. Under actual production operation, the complete online vision pipeline, from point-cloud acquisition by the camera to transmission of the final result, required approximately 2.04 s, including the mean registration-operator runtime of 531.03 ms. The scheduled production takt was 60 s, covering product picking and placement as well as interlayer-cardboard picking and placement. The complete vision pipeline therefore occupied approximately 3.40% of the scheduled takt time and completed well before the subsequent handling sequence required the result. Thus, the deployed vision system satisfied the timing requirement of the actual production line and did not constitute the production bottleneck.

To evaluate the method under one additional fixed production-line configuration separately from the 436-sample main experiment, an independent-line experiment was conducted using 48 samples of a previously unused heat-exchanger model with dimensions of 1.10 m × 0.79 m × 0.04 m. The corresponding model-specific template and dimensional prior were used, while the registration procedure and its settings were held fixed across all 48 samples without sample-specific adjustment. Relative to the reference registered templates, the mean translation error and nearest-neighbor RMSE were 5.21 mm and 4.34 mm, respectively. These results provide preliminary evidence that the proposed method can be applied to this additional known product model under the evaluated fixed production-line configuration.

#### 3.4.2. Component-Level Ablation Study

To isolate the contributions of the principal components of the proposed registration method, two ablated variants were evaluated using the same 436 production-line samples, manually annotated reference poses, preprocessing procedure, and parameter settings as those used in Section 3.4.1. In the first variant, the projection-extrema search was replaced by PCA-based principal-axis alignment. In the second variant, the tail-region depth-span criterion was retained for head–tail determination, but tail-edge anchoring was replaced by global centroid alignment between the incomplete observation and the complete CAD template. This variant, therefore, isolates the contribution of the longitudinal anchoring strategy without changing the head–tail decision. All remaining processing stages were kept unchanged.

In addition to the metrics defined in Section 3.4.1, the longitudinal error was calculated by projecting the centroid difference onto the longitudinal axis of the reference template:

(23) $$e_{L,i}=\left|{\left({\hat{c}}_{i}-c_{i}^{\mathrm{ref}}\right)}^{T}u_{i}\right|,$$

where $u_{i}$ denotes the unit longitudinal-axis vector of the reference template. Paired differences relative to the full method were evaluated using 10,000 cluster-bootstrap resamples, with the 44 production sets treated as the resampling units. All three configurations returned computational outputs for all 436 samples. The results are summarized in Table 9.

In Ablation A, replacing the projection-extrema search with PCA increased, the mean translation error from 5.21 mm to 42.31 mm and the mean yaw error from 0.23° to 7.42°. Relative to the full method, the paired increase in mean translation error was 37.11 mm, with a 95% confidence interval (CI) of [25.84, 49.62] mm. The corresponding increase in mean yaw error was 7.18°, with a 95% CI of [5.26°, 9.40°]. These results indicate that the projection-extrema search provides more stable planar orientation and coarse alignment than PCA when the observed surface is spatially incomplete.

In Ablation B, replacing tail-edge anchoring with global centroid alignment, the mean longitudinal error increased from 4.37 mm to 71.08 mm, and the mean translation error increased from 5.21 mm to 71.40 mm. The paired increases were 66.71 mm (95% CI: [50.75, 83.58] mm) and 66.20 mm (95% CI: [50.32, 83.57] mm), respectively. The mean yaw error remained essentially unchanged because the projection-extrema search and its short-side dimension verification were retained in this variant. The substantial increase in longitudinal error demonstrates that the centroid of the incomplete observation is strongly affected by the spatial distribution of the missing points, whereas the observed tail edge provides a more stable longitudinal reference.

Overall, the ablation results show that the projection-extrema search and tail-edge anchoring perform distinct and complementary functions: the former stabilizes planar orientation, whereas the latter suppresses longitudinal centroid bias under spatially nonuniform missingness.

**Figure 24:**
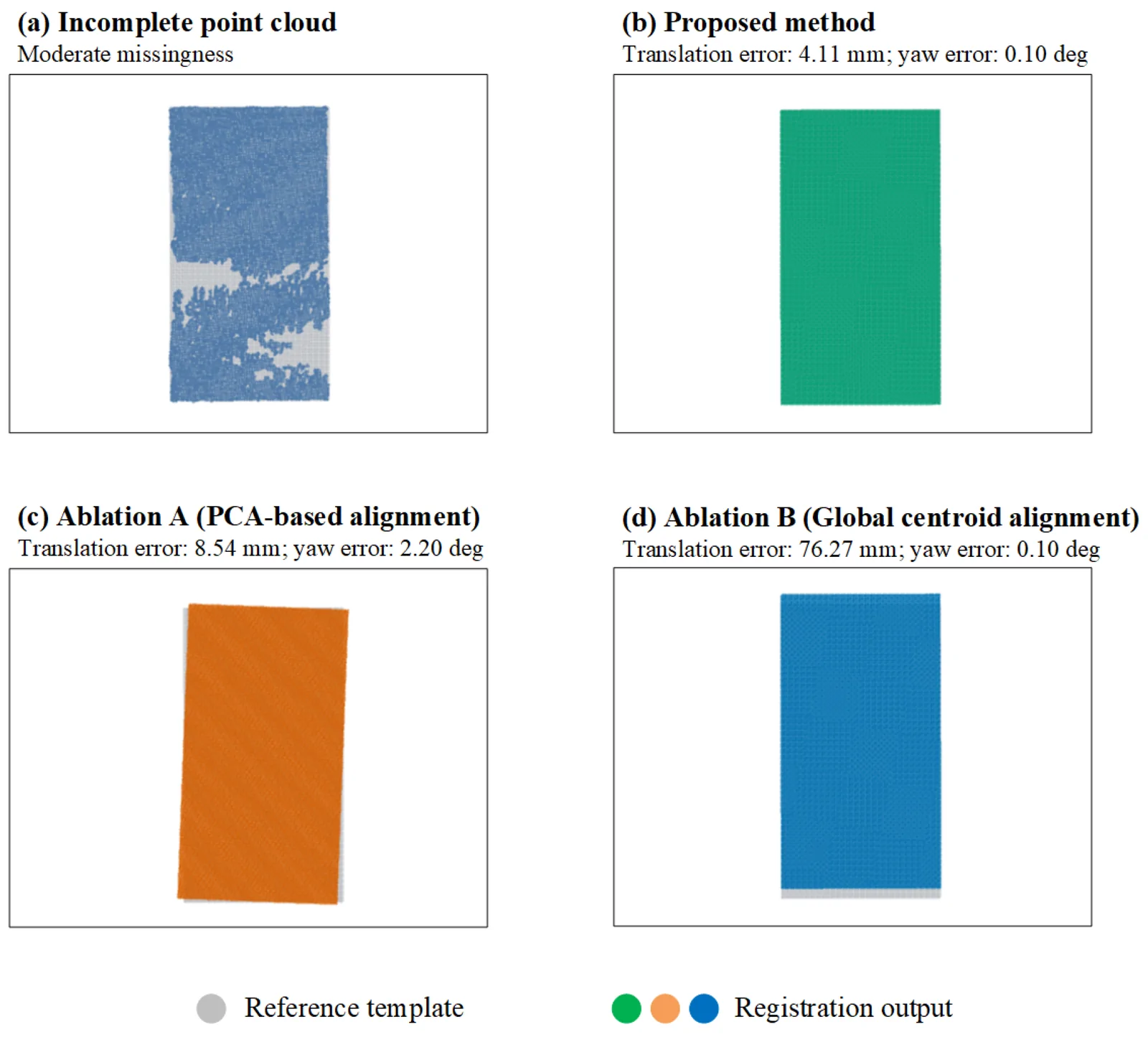
Qualitative component-level ablation results for a representative moderate-missingness sample: (**a**) incomplete point cloud; (**b**) proposed method; (**c**) Ablation A (PCA-based alignment); and (**d**) Ablation B (global centroid alignment). Gray points represent the reference template, and colored points represent the registration output (green: proposed method; orange: Ablation A; blue: Ablation B).

Figure 24 provides a qualitative visualization for a representative case with moderate missingness. The figure shows that the full method produces an output consistent with the reference template, whereas the PCA-based and centroid-based variants retain substantial orientation or longitudinal discrepancies.

#### 3.4.3. Parameter Sensitivity Analysis

The angular search resolution Δθ determines the sampling interval used in the projection-extrema-based coarse registration and may affect both orientation estimation and search cost. To evaluate the sensitivity of the proposed method to this parameter, Δθ was set to 0.1°, 0.25°, 0.5°, 1.0°, and 2.0°, corresponding to 1801, 721, 361, 181, and 91 candidate orientations, respectively, over the search interval of [−90°,90°]. All other registration parameters were kept unchanged, and the same 436 production-line samples and the evaluation metrics defined in Section 3.4.1 were used for every setting.

As shown in Table 10, the registration accuracy remained stable when the search step varied from 0.1° to 1.0°. Reducing the step below 0.5° substantially increased the number of candidate orientations, yet neither the mean translation error nor the mean nearest-neighbor RMSE decreased substantially. When the step was increased to 2.0°, the two metrics increased to 5.393 mm and 2.187 mm, respectively, indicating that an excessively coarse resolution increases the angular discretization error. Therefore, Δθ=0.5° was retained as a practical compromise between registration accuracy and search cost in the subsequent experiments.

#### 3.4.4. Robustness to Controlled Tail-Region Missingness

The proposed reverse-registration strategy uses the tail region as the longitudinal anchoring area. To quantitatively examine the robustness of this region under point-cloud missingness, 100 samples were randomly selected from the complete dataset of 436 samples. The selected subset covered all five product models. Five experimental conditions were considered. B0 denotes the original point cloud without artificial degradation. R15 and R30 simulate spatially scattered missing data by randomly removing tail-region information at target ratios of 15% and 30%, respectively. T15 and T30 simulate continuous tail occlusion by sequentially removing occupied grid rows from the physical tail toward the product interior at the same target ratios. B0, T15, and T30 were evaluated once for each sample, whereas R15 and R30 were independently repeated five times for each sample. A total of 1300 registration trials were therefore evaluated.

**Figure 25:**
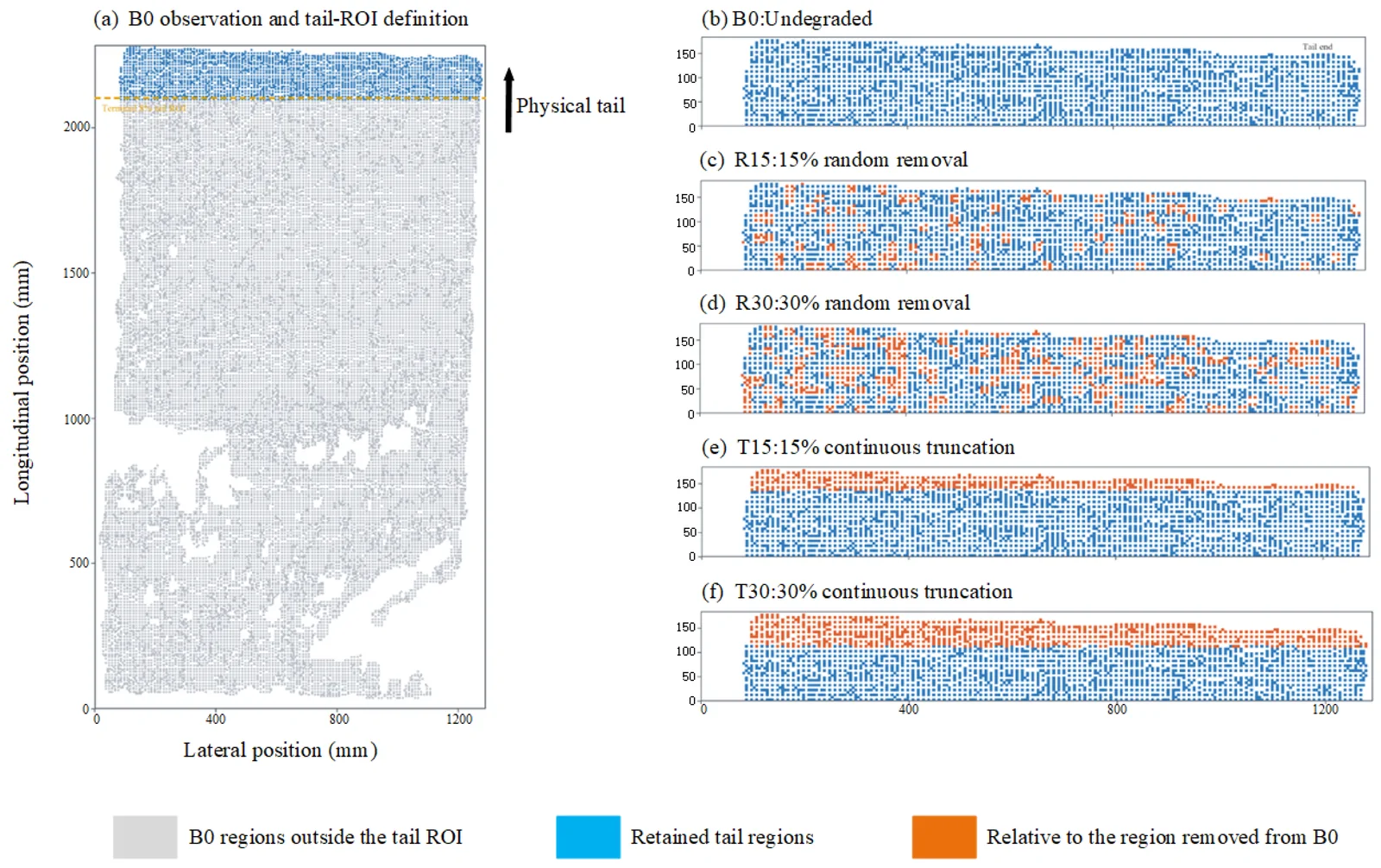
Construction of the controlled tail-region degradation conditions for a representative sample: (**a**) B0 observation and tail ROI definition; (**b**) undegraded tail region under B0; (**c**) R15; (**d**) R30; (**e**) T15; and (**f**) T30. Gray cells represent B0 regions outside the tail ROI, blue cells represent retained tail regions and orange cells represent the regions removed from B0.

Figure 25 illustrates the spatial effects of the five degradation conditions. Random removal reduces the local coverage of the tail region while retaining its overall longitudinal extent, whereas continuous truncation progressively shifts the observable tail boundary from the physical tail toward the product interior.

For each sample, the corresponding registered reference template was used to establish a local two-dimensional coordinate system. The projection of the global positive Y-direction onto the template plane was defined as the physical tail direction, and the terminal 8% of the template length was taken as the tail ROI. The reference template and product point clouds were projected onto the same local plane and discretized using a common grid origin and a fixed grid size of 5 mm. Only the template tail-ROI cells covered by the observed product point cloud were counted as effective tail information. The relative tail-information retention ratio under degradation condition *c* was defined as

(24) $$R_{i,c}=\frac{N_{i,c}}{N_{i,B0}}\times 100\%$$

where $N_{i,c}$ is the number of reference-template tail cells covered by sample *i* under condition *c*, and $N_{i,B0}$ is the corresponding value of the undegraded sample. Thus, the B0 retention ratio of each sample was normalized to 100%.

A registration trial was considered computationally completed when the proposed procedure returned a registration output. A completed trial was counted as a successful registration only when the head–tail orientation was also correctly determined. Therefore, a trial that produced an output but assigned the wrong head–tail orientation was counted as a failure in the success-rate calculation, while its numerical errors were still retained for the error analysis. The longitudinal error follows Equation (23), whereas the translation error and nearest-neighbor RMSE follow the definitions in Section 3.4.1. The values reported below are averages over the corresponding trials.

As shown in Table 11, random removal reduced local point coverage but preserved the overall longitudinal extent and distributed depth-gradient cues of the tail, allowing the registration procedure to remain stable under both random-degradation conditions.

Continuous truncation caused substantially larger longitudinal and translation errors than random removal because it directly displaced the observed tail boundary used for longitudinal anchoring. Under T30, all 100 trials completed computationally and produced registration outputs. However, 10 trials were assigned an incorrect head–tail orientation, resulting in 90 successful registrations out of 100 trials. These 10 trials were counted as failures in the successful-registration rate, but were retained in the calculation of the mean longitudinal error, mean translation error, and nearest-neighbor RMSE. Consequently, the T30 error statistics reported in Table 11 are calculated over all 100 trials rather than only the 90 successfully oriented trials. Nine of the 10 failures occurred in the two-column product variants. Specifically, the failure rate was 16.98% (9/53) for the two-column products, compared with 2.13% (1/47) for the three-column products. The stepped tail feature is approximately 60 mm long for the two-column products, whereas it is approximately 140 mm long for the three-column products. Consequently, continuous occlusion from the physical tail is more likely to remove a substantial portion of the discriminative stepped geometry in the two-column products, weakening the depth-span difference between the two longitudinal ends and increasing the probability of an incorrect head–tail decision. Under the evaluated fixed-camera acquisition conditions, the tail-anchoring strategy maintained correct head–tail determination under the B0, R15, R30, and T15 conditions, but became more sensitive to severe continuous occlusion under T30, particularly for products with a relatively short stepped tail feature.

#### 3.4.5. Comparison with Baseline Methods

Five representative baselines were evaluated on the same 436 incomplete point clouds: PCA + ICP, PCA + GICP, FPFH–RANSAC + ICP, KFPCS + ICP, and NDT + ICP. Together, they cover principal-axis initialization, local iterative refinement, descriptor-based global alignment, congruent-set matching, and distribution-based registration. For every sample, all methods used the same preprocessed incomplete observation, the corresponding model-specific complete CAD template, and the same manually annotated reference pose.

The parameter ranges were first examined in small pilot experiments using a subset drawn from the same 436-sample data pool. The relatively stable settings identified in these pilot experiments were then frozen and applied to all 436 samples without adjustment for an individual sample, product model, missing-data group, or failed trial. The initialization and fixed parameter settings are summarized in Table 12. Because the pilot subset was drawn from the evaluation pool, parameter selection and final evaluation were not independent; this may introduce dataset-specific selection bias into the reported baseline comparisons. The comparative results are therefore conditional on the selected parameter settings and the evaluated dataset. In particular, the statistical comparisons and confidence intervals reported below are conditional on the selected parameter settings and do not account for the uncertainty arising from parameter selection; they should therefore be interpreted as comparisons under a fixed, pre-specified configuration rather than as parameter-selection-independent estimates of generalization performance.

PCA + ICP and PCA + GICP used a 20-mm voxel size, a 160-mm maximum correspondence distance, 80 maximum iterations, and transformation and fitness thresholds of $10^{-8}$. FPFH–RANSAC used a 20-mm voxel size, normal and feature radii of 50 and 100 mm, respectively, 5000 maximum RANSAC iterations, three samples per hypothesis, correspondence randomness of five, a similarity threshold of 0.80, a minimum inlier fraction of 0.15, and a 200-mm correspondence distance. KFPCS used a 20-mm voxel size, an approximate overlap of 0.40, δ=30 mm, 200 source samples, 100 RANSAC base-selection attempts, and a 2-s global-search time budget. NDT used a 40-mm voxel size, a 100-mm resolution, a 50-mm step size, a transformation threshold of $10^{-6}$, and 35 maximum iterations. The ICP refinement after FPFH–RANSAC, KFPCS, and NDT used an 80-mm maximum correspondence distance, 50 maximum iterations, and transformation and fitness thresholds of $10^{-8}$.

For the two PCA-based baselines, the hypothesis with the lowest final correspondence fitness was retained. FPFH–RANSAC + ICP and KFPCS + ICP used the transformations returned by their respective global stages as the initial estimates for ICP. NDT + ICP evaluated four centroid-translated yaw hypotheses and passed the converged hypothesis with the lowest NDT fitness to ICP. The proposed method used its deterministic projection-extrema and tail-anchoring procedure; its initialization and parameter settings are described in Section 2.2.3 and in the preceding parameter-sensitivity analysis. No method received manual per-sample initialization.

Numerical convergence and valid output were recorded separately. Numerical convergence indicates that a solver satisfied its algorithm-specific stopping condition before reaching its iteration or time limit. A valid output indicates that the complete program returned a finite, rigid transformation and an evaluable registered point cloud. For PCA + ICP, a hypothesis was discarded when fewer than 20 valid correspondences remained; otherwise, its final finite estimate could be retained at the iteration limit. For the other baselines, failure of the global stage to return a finite converged hypothesis was recorded as a failed output and the local refinement was not used. A non-finite final transformation or a non-evaluable registered cloud was also treated as a failure.

PCA + ICP satisfied its epsilon-based stopping criterion for 270 of the 436 samples; the remaining 166 samples reached the iteration limit but returned finite, evaluable estimates. PCA + GICP, KFPCS + ICP, and NDT + ICP reported numerical convergence for all 436 samples. FPFH–RANSAC + ICP returned 104 converged global-and-local outputs, whereas its global RANSAC stage did not provide a valid initial transformation for the other 332 samples. The proposed method returned finite outputs for all 436 samples.

Because all methods were evaluated on the same observations, the statistical comparisons of registration errors were conducted on a paired basis. For each baseline, the sample-wise differences in translation error and nearest-neighbor RMSE relative to the proposed method were calculated as the baseline error minus the proposed-method error. The 95% confidence intervals of the mean paired differences were estimated using 10,000 cluster-bootstrap resamples, with the 44 production sets treated as the resampling units to preserve within-set dependence. Statistical significance was assessed using two-sided Wilcoxon signed-rank tests on the set-level mean paired differences. The resulting *p*-values were adjusted using the Holm procedure across the ten baseline–metric comparisons, with an adjusted p<0.05 considered statistically significant.

PCA + ICP, PCA + GICP, KFPCS + ICP and NDT + ICP were compared with the proposed method using all 436 common valid outputs from 44 production sets. The FPFH–RANSAC + ICP error comparison was restricted to its 104 common valid outputs from 40 production sets and was therefore interpreted conditionally together with its valid-output rate. The descriptive results and paired statistical comparisons are summarized in Table 13 and Table 14, respectively.

**Figure 26:**
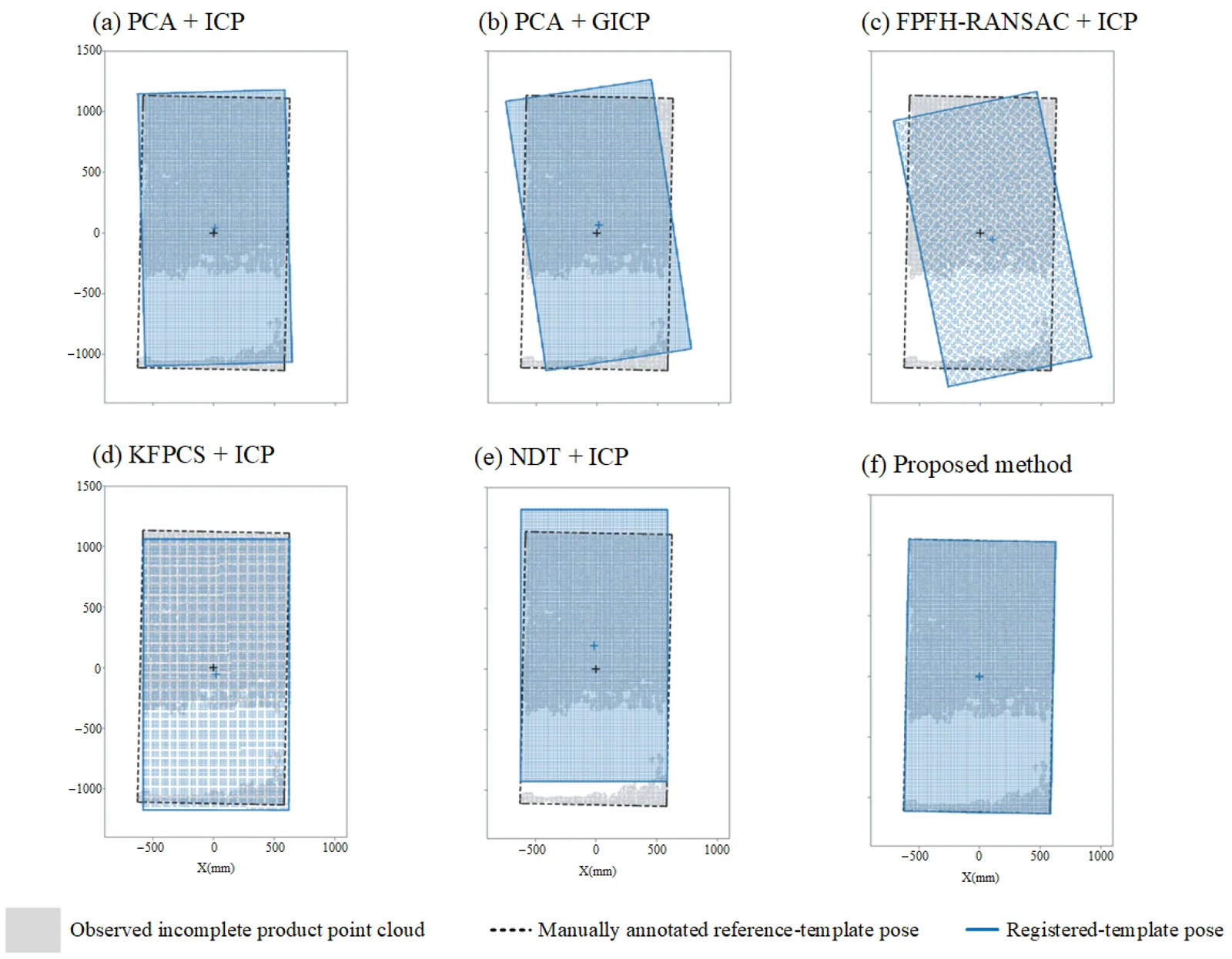
Qualitative registration comparison for the same severe-missingness observation and corresponding complete CAD template: (**a**) PCA + ICP; (**b**) PCA + GICP; (**c**) FPFH-RANSAC + ICP; (**d**) KFPCS + ICP; (**e**) NDT + ICP; and (**f**) the proposed method. Gray points represent the observed incomplete product point cloud, the black dashed boundary represents the manually annotated reference-template pose, and the blue points and boundary represent the registered-template pose.

All mean paired differences were positive, and all 95% cluster-bootstrap confidence intervals excluded zero. After Holm correction, the reductions in both translation error and nearest-neighbor RMSE were statistically significant for all five baseline comparisons (all adjusted p<0.001). For PCA + ICP, PCA + GICP, KFPCS + ICP, and NDT + ICP, the set-level mean differences were positive in all 44 production sets. For FPFH–RANSAC + ICP, they were positive in all 40 production sets containing valid outputs. A qualitative comparison is provided in Figure 26.

The results consistently show lower registration errors for the proposed method under the evaluated fixed parameter settings and production-line conditions. The low valid-output rate and comparatively large errors of FPFH–RANSAC + ICP are consistent with the limited distinctiveness of local features in the evaluated point clouds. The repeated fin structures generate similar local geometric neighborhoods, while reflection-induced missing regions disturb normal estimation and FPFH descriptor construction; RANSAC may therefore return an incorrect global pose hypothesis that cannot be corrected by the subsequent local ICP refinement. By using the prior product geometry, projection extrema, and a tail-region constraint, the proposed method avoids direct dependence on repeatable local descriptors and extensive complete overlap under the evaluated production-line conditions.

### 3.5. End-to-End Robotic Loading Performance

To evaluate the integrated system under actual production operation, 30 complete pallet-level runs covering four product models and two operating stations were analyzed. Each run began when a loaded pallet entered the operating station and ended after the products and interlayer cardboard had been handled. The recorded data included the RGB images, depth images, point clouds, and control messages generated during each operation. Normal empty-pallet records were excluded from the target-handling count, whereas abnormal empty-pallet detection attempts were retained separately to evaluate the reliability of production-cycle termination. A valid visual output was defined as a target pose that passed the geometric and safety-validity checks and was subsequently used to complete the associated handling operation.

A product-coordinate validity alarm was issued when the estimated product position lay outside the station-specific admissible workspace

$$\Omega_{\mathrm{adm}}=\Omega_{\mathrm{reach}}\cap \Omega_{\mathrm{station}}\cap \Omega_{\mathrm{collision}-\mathrm{free}},$$

where $\Omega_{\mathrm{reach}}$ is the reachable workspace of the robot, $\Omega_{\mathrm{station}}$ is the effective operating region of the corresponding station, and $\Omega_{\mathrm{collision}-\mathrm{free}}$ excludes the collision-risk boundaries associated with the gripper, receiving transfer mechanism, and surrounding equipment. An estimated target position $\hat{p}$ was accepted only if $\hat{p}\in \Omega_{\mathrm{adm}}$. Otherwise, robot motion was inhibited and scene reacquisition was triggered. An empty-pallet detection alarm was recorded when the system did not correctly confirm the terminal empty-pallet state. Multiple consecutive alarm signals associated with the same target or terminal-state check were merged into one abnormality episode. The end-to-end results are summarized in Table 15.

Nine target-handling operations generated product-coordinate validity alarms because the estimated positions violated the admissible-workspace constraints. These outputs were rejected before robot motion, and all nine targets were successfully localized and handled after one scene reacquisition.

Two additional abnormality episodes occurred during empty-pallet detection. They generated two and three consecutive alarm signals, respectively, before reacquisition confirmed the empty-pallet state; both pallet-level runs then terminated normally. Thus, all 11 recoverable visual-recognition abnormalities were resolved automatically without manual intervention, and no rejected visual output caused an erroneous grasp. Twenty-one pallet-level runs proceeded without an alarm, while the nine runs containing an abnormality were automatically recovered. This distinction separates first-attempt localization reliability from final task completion under the evaluated conditions.

## 4. Conclusions

This paper developed a 3D vision-based localization and guidance system for automated loading of large finned-tube heat exchangers. Its main methodological contribution is a model-specific integration of projected-area yaw estimation and lateral alignment, tail-constrained head–tail determination and longitudinal anchoring, and inverse mapping of a complete CAD template. The integrated procedure uses the remaining reliable global and tail-region geometry to localize heat exchangers affected by structured reflection-induced missingness. Plane correction and dual-branch interlayer-cardboard extraction provide the corrected target point clouds required by the integrated loading workflow.

In the 436-sample main registration experiment, the method achieved a mean translation error of 5.21 mm, a nearest-neighbor RMSE of 1.98 mm, and an average registration-operator runtime of 531.03 ms. Component-level ablation showed that replacing projection-extrema alignment with PCA and replacing tail-edge anchoring with global centroid alignment increased the mean translation error to 42.31 mm and 71.40 mm, respectively. In the end-to-end evaluation, the first-attempt target-localization success rate was 98.53%, and all 612 robotic handling operations across 30 pallet-level runs were completed without an unrecovered failure under the evaluated conditions.

Although the additional experiment demonstrated successful application to one previously unused product model on another production line, this evaluation covered only one additional model. Therefore, the applicability of the proposed method to structurally different heat-exchanger families and other industrial components with substantially different geometries, materials, or reflection patterns requires further investigation. For heat exchangers of different sizes and models, the surface geometry and reflection-induced missing regions may vary in production. Selecting local high-confidence features and dynamically adjusting matching and downsampling thresholds could further improve the applicability of the algorithm to nonstandard products. Adaptive feature-search and dynamic-threshold mechanisms for multiple heat-exchanger variants remain subjects for future research.

The reported performance is limited to the evaluated fixed production stations, installed camera configurations, normal production illumination, known heat-exchanger models, and white or brown cardboard materials. Camera pose and illumination were not systematically varied; therefore, the reported robustness primarily concerns product-model, stacking-configuration, stack-height, and point-cloud-missingness variations within the calibrated operating conditions. Although the geometry-based fallback reduces dependence on color information when the primary branch fails, deployment after changes in camera mounting, illumination, or cardboard appearance requires recalibration of the station ROI and reference-plane correction, revalidation of the color and geometric acceptance parameters, and verification of registration performance using representative samples from the new operating condition. Table 3 summarizes which settings remain fixed across product models and which must be recalibrated for a new model, station, or cardboard material, and thus defines the practical boundary for transferring the method to another production line without retuning.

## Figures and Tables

**Table 1:** Geometric features and filtering criteria for the cardboard mask.

Geometric Feature	Formula	Filtering Function	Threshold Range
Connected component area *A*	Total number of pixels	Remove abnormal area regions	40,000 < A < 80,000
Aspect ratio $R_{lw}$	L/W	Exclude interference regions	$1.5<R_{lw}<4$
Rectangularity $R_{r}$	$A/A_{rect}$	Exclude irregular regions	$R_{r}>0.9$

Note: *L* and *W* denote the long and short sides of the bounding rectangle, respectively, and $A_{rect}$ denotes the area of the bounding rectangle.

**Table 2:** Dimensions of the main product models.

Name	Length (mm)	Width (mm)	Thickness (mm)	Weight (kg)
SMMS-U 2-column	1355	1220	43.3	25
SMMS-U 3-column	1355	1220	64.9	37
SMMS-U 2-column long	2000	1220	43.3	37
CR SMMS-U 2-column	2300	1220	43.3	42
CR SMMS-U 3-column	2300	1220	64.9	63

**Table 3:** Configuration scope of the principal parameters: fixed versus model-, station-, or material-specific settings.

Parameter/Input	Scope	Setting or Determining Factor
Downsampling and outlier filtering	Global (fixed)	8-mm voxel grid
Projection search	Global (fixed)	Search interval: [−90°,90°]; angular step: 0.5°
CAD template and dimensional priors	Model-specific	Product geometry and nominal dimensions
Tail threshold $T_{d}$	Model-specific	Product-column configuration
ROI and reference-plane correction	Station-specific	Camera installation and workstation geometry
Cardboard-detection criteria	Material-specific	Illumination, cardboard appearance, dimensions, and expected cardboard count

Note: Settings marked “Global (fixed)” are identical across the evaluated product models and stations. Settings that depend on the product model, station, or cardboard material are configured before operation and revalidated whenever the corresponding condition changes (a new product model; camera remounting or a station change; or a change in illumination, cardboard appearance, or the expected cardboard count).

**Table 4:** Plane-correction results for ten consecutive stack layers.

Layer	Robust Z Span Before (mm)	Robust Z Span After (mm)	Inclination Before Correction (°)	Inclination After Correction (°)	Plane RMSE (mm)
1	58.83	17.09	2.507	0.508	2.32
2	54.30	14.64	2.243	0.239	2.42
3	54.99	16.24	2.350	0.365	2.85
4	53.77	14.59	2.190	0.191	2.61
5	52.36	14.65	2.196	0.192	2.67
6	52.39	14.39	2.172	0.169	2.75
7	50.95	13.48	2.077	0.073	2.59
8	54.07	17.13	2.234	0.269	3.39
9	52.66	15.62	2.118	0.155	3.15
10	56.78	17.53	2.253	0.248	3.17
Mean ± SD	54.11±2.32	15.54±1.39	2.234±0.122	0.241±0.122	2.79±0.35

**Table 5:** Performance of the color-based branch and the geometry-based fallback.

Station	Samples	Color Correct (%)	Fallback Triggered (%)	Fallback Success	Final Correct (%)
Station 1	62	58 (93.55)	4 (6.45)	4/4	62 (100.00)
Station 2	67	64 (95.52)	3 (4.48)	3/3	67 (100.00)
Overall	129	122 (94.57)	7 (5.43)	7/7	129 (100.00)

**Table 6:** Registration accuracy of the proposed algorithm under different levels of missing data.

Missing-Data Level	Number of Samples	Mean Translation Error [95% CI] (mm)	Translation ErrorMedian/P95 (mm)	Mean Nearest-NeighborRMSE [95% CI] (mm)	Mean YawError (°)
Low (<15%)	193	5.12 [4.34, 5.99]	4.06/12.36	1.89 [1.74, 2.05]	0.30
Moderate (15%–30%)	199	5.49 [4.84, 6.17]	4.56/12.12	2.04 [1.95, 2.13]	0.18
Severe (>30%)	44	4.28 [3.80, 4.78]	3.93/7.51	2.07 [1.93, 2.19]	0.20
Overall	436	5.21 [4.76, 5.68]	4.36/11.86	1.98 [1.89, 2.06]	0.23

**Table 7:** Registration accuracy stratified by product model and missing-data level.

Missing-Data Level	Product Model	Number of samples	Mean Translation Error (mm)	Mean Nearest-Neighbor RMSE (mm)
Low	1	9	4.42	1.72
Low	2	2	3.26	1.88
Low	3	120	5.07	1.89
Low	4	21	5.30	1.80
Low	5	41	5.41	1.98
**Low subtotal**	–	**193**	**5.12**	**1.89**
Moderate	1	77	6.11	2.12
Moderate	2	17	7.51	1.99
Moderate	3	18	5.97	2.00
Moderate	4	0	–	–
Moderate	5	87	4.46	1.99
**Moderate subtotal**	–	**199**	**5.49**	**2.04**
Severe	1	11	4.31	2.12
Severe	2	1	7.21	2.17
Severe	3	7	3.61	1.90
Severe	4	0	–	–
Severe	5	25	4.33	2.09
**Severe subtotal**	–	**44**	**4.28**	**2.07**
**Overall**	–	**436**	**5.21**	**1.98**

Note: bold rows are the subtotals for each missing-data level, and the “Overall” row is the grand total over all samples.

**Table 8:** Stage-level computational cost for a representative low-missingness sample.

Algorithm Stage	Runtime (ms)	Proportion of Operator Runtime (%)
Input point-cloud conversion	1.55	0.28
Fixed reference-plane correction	1.09	0.19
Projection-extrema search	530.00	94.65
Head–tail determination and orientation	2.38	0.43
Tail anchoring	3.03	0.54
Template normalization	17.39	3.11
Inverse template transformation	1.76	0.31
Output point-cloud conversion	2.75	0.49
Complete registration operator	559.96	100.00

**Table 9:** Component-level ablation results on the 436 production-line samples.

Configuration	Component Setting	Mean Longitudinal Error (mm)	Mean Translation Error (mm)	Mean Yaw Error (°)	Mean NN RMSE (mm)
Proposed method	PEA + TEA	**4.37**	**5.21**	**0.23**	**1.98**
Ablation A	PCA + TEA	39.38	42.31	7.42	13.55
Ablation B	PEA + GCA	71.08	71.40	0.24	11.68

Note: PEA denotes projection-extrema alignment; PCA denotes PCA-based principal-axis alignment; TEA denotes tail-edge anchoring; and GCA denotes global-centroid alignment. The tail-region depth-span criterion for head–tail determination was retained in all configurations. Each ablated variant replaced only the indicated component, while all other processing stages and parameters remained unchanged. Bold values indicate the best results.

**Table 10:** Sensitivity of registration accuracy to the projection-search resolution.

Search Step (°)	Candidate Orientations	Mean Translation Error (mm)	Mean Nearest-Neighbor RMSE (mm)
0.10	1801	5.138	1.859
0.25	721	5.159	1.901
0.50	361	5.205	1.977
1.00	181	5.305	2.080
2.00	91	5.393	2.187

**Table 11:** Performance under controlled tail-region degradation conditions.

Condition	Tail Retention (%)	Success Rate (%)	Mean Longitudinal Error (mm)	Mean Translation Error (mm)	Mean RMSE (mm)
B0	100.00	100	4.36	5.34	1.91
R15	85.64	100	4.73	5.69	2.06
R30	71.37	100	5.19	6.26	2.42
T15	85.01	100	21.61	22.15	2.95
T30	70.00	90	37.26	38.22	4.89

Note: Success rates for B0, T15, and T30 were calculated from 100 trials; those for R15 and R30 were calculated from 500 trials because each of the 100 samples was independently degraded and evaluated five times.

**Table 12:** Fixed configurations of the baseline registration methods.

Method	Initialization	Local Refinement	Termination and Failure Handling
PCA + ICP	Four sign-consistent PCA-axis hypotheses; centroid-aligned translation	ICP	Epsilon-based convergence or the 80-iteration limit; hypotheses with fewer than 20 valid correspondences were discarded.
PCA + GICP	The same four PCA hypotheses and centroid alignment	GICP	Epsilon-based convergence or the 80-iteration limit; a non-finite final output was recorded as a failure.
FPFH–RANSAC + ICP	FPFH prerejective RANSAC transformation	ICP	Failure was recorded when RANSAC returned no finite converged transformation.
KFPCS + ICP	KFPCS global transformation	ICP	A 2-s global-stage limit was imposed; a non-finite global or final output was recorded as a failure.
NDT + ICP	Four centroid-aligned yaw hypotheses separated by 90°	ICP	NDT convergence or the 35-iteration limit; absence of a finite converged hypothesis was recorded as a failure.

**Table 13:** Descriptive comparison with representative baseline registration methods under fixed parameter settings.

Method	Valid Output Rate (%)	Mean Translation Error (mm)	Mean Nearest-Neighbor RMSE (mm)	Number of Valid Outputs
PCA + ICP	100.00	45.27	17.96	436
PCA + GICP	100.00	52.68	21.24	436
FPFH–RANSAC + ICP	23.85	290.93	127.23	104
KFPCS + ICP	100.00	56.16	17.05	436
NDT + ICP	100.00	56.69	17.82	436
Proposed method	100.00	**5.21**	**1.98**	436

Note: All methods processed the same 436 incomplete observations. The valid-output rate uses all 436 samples as the denominator, whereas the two error metrics use valid outputs only. Bold values indicate the best results.

**Table 14:** Paired statistical comparisons of baseline registration errors relative to the proposed method.

Method	Common Valid Samples/Sets	Mean Paired Difference [95% CI] (mm)	Holm-Adjusted *p*-Value
*(a) Translation error*			
PCA + ICP	436/44	40.07 [33.74, 47.12]	<0.001
PCA + GICP	436/44	47.48 [40.06, 55.83]	<0.001
FPFH–RANSAC + ICP	104/40	285.78 [235.51, 338.65]	<0.001
KFPCS + ICP	436/44	50.95 [43.14, 59.80]	<0.001
NDT + ICP	436/44	51.49 [45.22, 58.67]	<0.001
*(b) Nearest-neighbor RMSE*			
PCA + ICP	436/44	15.99 [14.15, 18.06]	<0.001
PCA + GICP	436/44	19.26 [17.11, 21.52]	<0.001
FPFH–RANSAC + ICP	104/40	125.24 [100.81, 152.11]	<0.001
KFPCS + ICP	436/44	15.07 [14.05, 16.10]	<0.001
NDT + ICP	436/44	15.84 [14.53, 17.26]	<0.001

**Table 15:** End-to-end performance of the integrated robotic loading system.

Metric	Result
Complete pallet-level runs	30
Successful product-loading operations	308/308 (100.00%)
Successful cardboard-removal operations	304/304 (100.00%)
Successful robot-handling operations	612/612 (100.00%)
First-attempt target-localization success	603/612 (98.53%)
Product-coordinate validity alarm episodes	9
Empty-pallet detection alarm episodes/signals	2/5
Automatically recovered abnormality episodes	11/11 (100.00%)
Pallet-level runs containing an abnormality	9/30 (30.00%)
Final pallet-level completion rate	30/30 (100.00%)

## Data Availability

The complete dataset is not publicly available because it was collected in an operating industrial production environment and contains product-related images and manufacturing-process information subject to commercial confidentiality restrictions. De-identified, non-confidential sample data and the associated acquisition and evaluation protocols are available from the corresponding author upon reasonable academic request, subject to the applicable confidentiality restrictions.
